# Harnessing microfluidic streak plate technique to investigate the gut microbiome of *Reticulitermes chinensis*

**DOI:** 10.1002/mbo3.654

**Published:** 2018-06-13

**Authors:** Nan Zhou, Yu‐Tong Sun, Dong‐Wei Chen, Wenbin Du, Hong Yang, Shuang‐Jiang Liu

**Affiliations:** ^1^ State Key Laboratory of Microbial Resources and Environmental Microbiology Research Center at Institute of Microbiology Chinese Academy of Sciences Beijing China; ^2^ University of Chinese Academy of Sciences Beijing China; ^3^ College of Life Science at Hebei University Baoding China; ^4^ School of Life Sciences at Central China Normal University Wuhan China

**Keywords:** bacterial diversity, cultivation, gut microbiome, microfluidic streak plate (MSP), *Reticulitermes chinensis*

## Abstract

The termite gut microbiome is a model system to investigate microbial interactions and their associations with host. For decades, extensive research with molecular tools and conventional cultivation method has been carried out to define the microbial diversity in termite gut. Yet, many bacterial groups of the termite gut microbiome have not been successfully cultivated in laboratory. In this study, we adapted the recently developed microfluidic streak plate (MSP) technique for cultivation of termite gut microbial communities at both aerobic and anaerobic conditions. We found that 99 operational taxonomic units (OTUs) were cultivable by MSP approach and 18 OTUs were documented first time for termite gut microbiota. Further analysis of the bacterial diversities derived by culture‐dependent MSP approach and culture‐independent 16S rRNA gene typing revealed that both methods have bias in recovery of gut microbiota. In total 396 strains were isolated with MSP technique, and potential new taxa at species and/or genus levels were obtained that were phylogenetically related to *Burkholderia*,* Micrococcus*, and *Dysgonomonas*. Results from this study indicate that MSP technique is applicable for cultivating previously unknown and new microbial groups of termite gut microbiota.

## 1 INTRODUCTION

The gut of termite harbors a dense and diverse microbiota of approximately 10^6–8^ bacterial cells (Breznak, 1982, 2000). This microbiota and their symbiosis with host are essential for the efficient digestion of lignocellulose in termite gut (Brune & Dietrich, 2015; Ohkuma, 2003; Warnecke et al., 2007). For several decades, the gut microbiome of termites has been attracting interest from microbiologists and biotechnologists (Breznak, 1982; Brune & Friedrich, 2000; Ohkuma & Kudo, 1996), since termite gut microbiome not only plays important roles in carbon turnover in the environment but also is potential sources of biochemical catalysts converting wood into biofuels (Warnecke et al., 2007). Wood‐feeding termites can digest up to 83%–85% of glucosyl and xylosyl residues from lignocellulose (Bignell, 2011). Termite gut microbiomes have been exploited for production of carboxylates from low‐value biomass (Ali et al., 2017; Auer et al., 2017; Ni & Tokuda, 2013; Watanabe & Tokuda, 2010) as well as to discover commercially important enzymes (Cibichakravarthy, Abinaya, & Prabagaran, 2017; Liu et al., 2011; Martin & Martin, 1978; Matsuura, Yashiro, Shimizu, Tatsumi, & Tamura, 2009). Culture‐independent 16S rRNA gene typing and metagenomic tools have been extensively used for description of the termite gut microbial community (Huang, Bakker, Judd, Reardon, & Vivanco, 2013; Ohkuma & Brune, 2011; Tarayre et al., 2015).

Compared to culture‐independent methods, the culture‐dependent method would better serve the purpose to investigate host‐microbe interaction or to recover valuable microbial products (including commercial enzymes) (Keller & Zengler, 2004; Stewart, 2012). However, cultivation of microbes from various samples including termite gut is often hindered as many microbes in nature are resistant to be cultivated in laboratory conditions (Amann, Ludwig, & Schleifer, 1995; Hongoh, 2011; Ohkuma & Brune, 2011). To overcome this obstacle and to cultivate as yet not cultivated microorganisms in laboratory, techniques of high throughput and mimic natural conditions have been developed, such as the high‐throughput culturing procedures that utilize the concept of extinction culturing (Colin, Goñiurriza, Caumette, & Guyoneaud, 2013; Colin, Goñi‐Urriza, Caumette, & Guyoneaud, 2015; Connon & Giovannoni, 2002), the microencapsulation (Keller & Zengler, 2004; Zhou, Liu, Liu, Ma, & Su, 2008) and the isolation chip (Ichip) (Nichols et al., 2010). Microfluidic devices (Ma et al., 2014; Park, Kerner, Burns, & Lin, 2011; Tandogan, Abadian, Epstein, Aoi, & Goluch, 2014) were also developed for highly parallel cocultivation of symbiotic microbial communities and isolating pure bacterial cultures from samples containing multiple species. The microfluidic streak plate (MSP) technique (Jiang et al., 2016) exploits the advantages of microfluidics to manipulate tiny volume of liquid at several to hundred nanoliters and generate microdroplets for microbial single‐cell isolation and cultivation. Superior to the conventional agar plate cultivation, the MSP approach enabled higher throughput of bacterial isolation and better coverage of rare species in community (Jiang et al., 2016).

*Reticulitermes chinensis* (Snyder) (Isoptera: Rhinotermitidae) is wood‐feeding lower termite. In this study, we continued our efforts to cultivate microbes from the gut of from this termite (Chen, Wang, Hong, Yang, & Liu, 2012; Fang, Lv, Huang, Liu, & Yang, 2015; Fang et al., 2016), and adapted the MSP technique for cultivation of gut microbiome at both aerobic and anoxic conditions. With the MSP method, 99 OTUs representing *Proteobacteria*,* Firmicutes*,* Actinobacteria*,* Bacteriodetes*,* Acidobacteria,* and *Verrucomicrobia* were obtained, and 396 bacterial isolates were successfully cultivated in pure cultures. Our results demonstrated that MSP method significantly increased the recovery of various microbial groups and many of them were documented for the first time from termite gut.

## 2 MATERIALS AND METHODS

### 2.1 Termite cultivation and retrieving gut microbiota

The termite *Reticulitermes chinensis* colonies were collected and transferred to laboratory, and were maintained in glass containers on a diet of pinewood and water. Only worker termites were used in this study. The termite's surface was washed three times with 70% ethanol, rinsed with distilled water and blotted dry on sterilized filter papers. The guts from 40 termites were removed aseptically with fine‐tipped forceps onto a sterilized glass slide and the gut microbiota were squeezed out of the guts and were transferred into a tube with 1mL of PBS buffer (PBS buffer, g/L: NaCl, 8.00; KCl, 0.20; Na_2_HPO_4_.12H_2_O, 3.58; KH_2_PO_4_, 0.24; pH 7.2). The gut microbiota suspension in the PBS buffer was used subsequently for cell separation and cultivation.

### 2.2 Operation of microfluidic droplet arrays

Microfluidic streak plate (MSP) was operated according to previously described (Jiang et al., 2016), except that the automated dish driver and the microfluidic device were setup in an anaerobic chamber (ThermoScientific 1029). Droplets were arrayed onto surface‐modified Petri‐dish (Jiang et al., 2016), and about 3000 droplets were displayed onto the surface of 9‐cm Petri‐dish.

### 2.3 Dilution of gut microbiota samples and cultivation of microbes

Fivefold‐diluted (1/5) R2A medium (1/5 R2A, g/L: Yeast extract, 0.1; Peptone, 0.1; Casamino Acids, 0.1; Glucose, 0.1; Soluble starch, 0.1; Sodium pyruvate, 0.1; K_2_HPO_4_, 0.75; KH_2_PO_4_, 0.75; MgSO_4_·7H_2_O, 0.2; pH 7.2) was used as growth broth and for dilution of gut microbiota samples. In order to prepare samples for MSP, the gut microbiota suspension (see M&M section 1) was diluted with growth broth, either directly from the suspension or after three times washing with Cysteine‐reduced (1 g/L) PBS buffer (pH 7.2). The final concentration of diluted gut microbiota suspension was approximately 1 × 10^4–5^ cells/ml. This diluted suspension was used for separation and cultivation of the gut microbiota with the MSP method. Petri dishes with droplet arrays were incubated at 30°C under both aerobic and anaerobic condition. After 72 hr incubation, the droplets were individually transferred into 96‐well cell‐culture plates, each well contained 80 μL of 1/5 R2A medium. After another 72 hr of cultivation at 30°C, the growth of bacterial cells was monitored with a Microplate reader (Biotek SynergyHT). The grown cells were streaked on R2A agar plates, and all bacterial strains obtained were stored at 10°C in cold room until further tests.

### 2.4 Total DNA extraction, amplification of 16S rRNA genes, and DNA sequencing

Cells of termite gut samples and from MSP droplet arrays were collected by centrifugation. Metagenomic DNA was extracted with E.Z.N.A Meg‐Bind Soil DNA Kit (Omega Bio‐tek, GA, USA) using a KingFisher Flex Magnetic Particle Processor (Thermo Scientific, MA, USA). Extractions were performed according to Kit and instrument protocols. Purified DNA were used for 16S rRNA gene amplification with the PCR primers (targeted the V4 region) U515F (5′‐GTGCCAGCMGCCGCGGTAA‐3′) and 806R (5′‐GGACTACHVGGGTWTCTAAT‐3′) containing barcodes at the 5′ end of the front primer (Werner, Zhou, Caporaso, Knight, & Angenent, 2012). PCR reactions were proceeded in 50 μL volumes, each containing 1.5 μL of 10 μM forward and reverse primers, respectively, 25 μL of 2× KAPA HiFi HotStart ReadyMix (Kapa Biosystems, Inc., MA, USA), and up to 22 μL of purified DNA as template. The thermocycling was performed as follows: 30 cycles (98°C, 20 s; 54°C, 15 s; 72°C, 15 s) after an initial denaturation at 95°C for three min, following a final extension at 72°C for 60 s. Triplicate PCR products for each sample were purified using E.Z.N.A Gel Extraction Kit (Omega Bio‐Tek, Inc.) and then quantified using Qubit dsDNA HS Assay Kit (Invitrogen, CA, USA). Equal amounts of PCR products were mixed to produce equivalent sequencing depth from all samples. After purification using Agencount AMPure XP KIT, the pooled‐PCR products were used to construct a DNA library using NEB E7370L DNA Library Preparation Kit. The libraries were sequenced on an Illumina MiSeq 2500 platform at BGI GENE (Wuhan, China). Complete data with 250 bp reads had been submitted to the NCBI Short Read Archive database under accession No. SRP133587

The full length of 16S rRNA gene from each bacterial strain obtained in this study was amplified with the 27F and 1492R primers (Edwards, Rogall, Blöcker, Emde, & Böttger, 1989; Weisburg, Bars, Pelletier, & Lane, 1991). The 16S rRNA gene sequences of the isolates in this study have been deposited in GenBank databases under the accession numbers MG984070‐MG984092.

### 2.5 16S rRNA gene‐based metagenomic analysis and phylogenetic tree construction

The raw sequences were assigned to individual samples by their unique barcodes. The 16S rDNA primers and barcodes were then removed to generate pair‐end (PE) reads. Raw tags were then generated by merging PE reads with FLASH (Magoč & Salzberg, 2011), the raw tags were then filtered and analyzed using QIIME software package (Quantitative Insights Into Microbial Ecology) (Bokulich et al., 2013). Reads from all samples were quality filtered using an average quality value of 20 (Q20) during demultiplexing, sequences with a mean quality score 20 were excluded from analysis, and chimeras were also excluded. For species analysis, 16S rRNA sequences with ≥97% similarity were assigned to the same OTUs using Uparse v7.0.1001 (Edgar, 2013), and similarity hits below 97% were not considered for classification purpose. A representative sequence of each OTU was picked out and the taxonomic information was annotated using RDP classifier (version 2.2) (Wang, Garrity, Tiedje, & Cole, 2007) and GreenGene database (Desantis et al., 2006). Sequences obtained were compared with the published sequences in GenBank using Blast from NCBI (http://www.ncbi.nlm.nih.gov/BLAST).

The 16S rRNA sequences of all the published termite‐gut‐derived bacteria were mined from NCBI. The OTU sequences of MSP pool sample were blasted with the GenBank of NCBI and the 16S rRNA sequences of type species with the highest similarity to our OTUs were selected. Those sequences together with the extracted termite‐gut‐derived bacterial 16S rRNA gene sequences were used for the construction of phylogenetic tree. The OTUs from MSP pool samples (accession numbers MH152413‐MH152511), the 16S rRNA gene sequences of isolated strains (accession numbers MG984070‐MG984092) and the reference sequences (the accession number was available in phylogenetic tree) were aligned using ClustalW (Thompson, Gibson, & Higgins, 2002). Phylogenetic trees were constructed with MEGA6 package based on the alignments of sequences using Neighbor‐joining method with p‐distance. Bootstrap analysis with 1000 replicates was performed to determine the statistical significance of the branching order.

## 3 RESULTS

### 3.1 Termite gut microbial community revealed with MSP technique and comparison to metagenomic method

**Figure 1:**
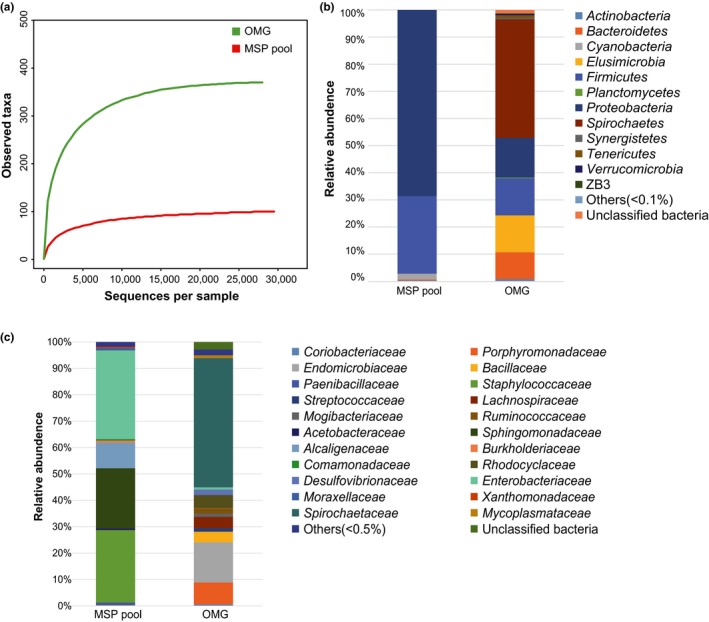
Rarefaction curves of 16S rDNA sequences of the samples (a), the relative abundances of the dominant Phylum in all samples indicated and the rest being labeled as “Others” (b) and the relative abundances of the dominant Families in all samples (c). Curves were calculated based on OTUs at 97% similarity

We sequenced both the partial 16S RNA gene of the original microbiota from gut sample (hereafter called OMG sample) and DNA extracted from the pooled droplets from cultured MSP plates (hereafter called MSP pool). A total of 38,056 and 37,137 Pair‐end reads were retrieved, and after filtering and removing potential erroneous sequences, a total of 28,422 and 29,778 effective tags were obtained from OMG sample and MSP pool, respectively. These sequences represented 58,200 taxon tags that covered 141 genera, 102 families, 57 orders, or 33 classes of 15 phyla. As shown in Figure 1a, the rarefaction curves of OMG and MSP pool reached plateau after 10,000 and 5000 sequences per sample, respectively, indicating that the sequencing depth was adequate to reflect the bacterial diversity in both samples. Data analysis showed that OMG sample had much higher OTU richness than the MSP samples, At the phylum level, the relative abundances of five phyla in OMG samples and two phyla in MSP pool sample were higher than 1% (Figure 1b, for details please see Tables S1, S2 and S3). To be specific, *Spirochaetes* (44.3%), *Proteobacteria* (14.7%), *Firmicutes* (13.9%), *Elusimicrobia* (13.8%), and *Bacteroidetes* (10.0%) were the top five phyla in the OMG sample, whereas *Proteobacteria* (69.9%) and *Firmicutes* (29.2%), were the top two phyla in the MSP pool sample. We found that six phyla (*Proteobacteria*,* Firmicutes*,* Bacteroidetes*,* Actinobacteria*,* Planctomycetes,* and *Verrucomicrobia*) presented in both OMG sample and MSP pool, suggesting members of those phyla were culturable with the MSP technique when the 1/5 R2A medium was used. Furthermore, *Proteobacteria* and *Firmicutes* were among the dominant phyla in both OMG sample and MSP pool, indicating they were well represented in the MSP pool. Significant differences were also observed: the phyla of *Acidobacteria*,* Fusobacteria*,* Nitrospirae*, and *Thermi* were only observed with MSP pool, whereas the phyla of *Spirochaetes*,* Elusimicrobia*,* Synergistetes*,* Tenericutes,* and ZB3 were only observed with OMG sample. When analyzed at Family level (Figure 1b), 19 of the total 102 families were found in both OMG sample and MSP pool and they accounted for 31.1% of the total taxon tags. The *Spirochaetaceae* (44.3%), *Endomicrobiacea* (13.8%), *Porphyromonadaceae* (7.5%), *Rhodocyclaceae* (4.5%), and *Lachnospiraceae* (3.8%) were the dominant families of OMG sample, whereas the Enterobacteriaceae (33.6%), *Staphylococcaceae* (27.4%), and *Sphingomonadaceae* (22.8%), *Alcaligenaceae* (9.7%)were the dominant families in MSP pool (Figure 1c).

### 3.2 Identification of yet‐to‐be cultured microbial OTUs/taxa from MSP pool

**Figure 2:**
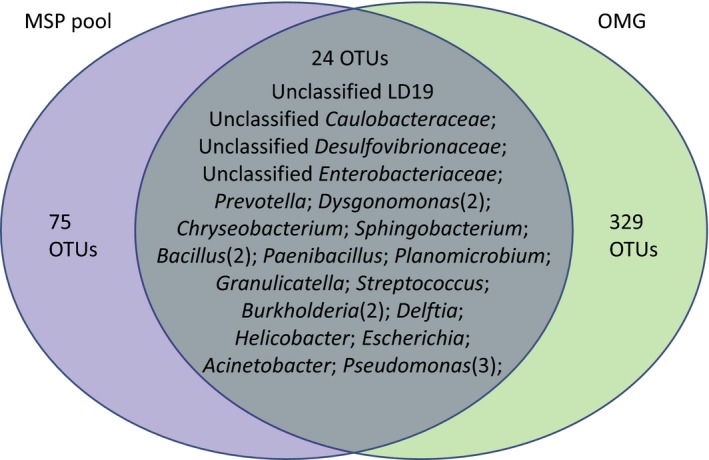
Venn diagram of OTUs in the two samples. Unique and shared OTUs in the two samples are based on 97% similarity. The numbers inside the diagram indicate the numbers of OTUs

With a cutting edge of 97% sequence similarity, 99 and 353 OTUs from MSP pool and OMG sample, respectively, were recognized. Venn diagram showed that OMG and MSP shared 24 OTUs, but more OTUs were uniquely in either MSP pool or OMG sample (Figure 2). This is one more example representing that the microbial diversities was differentially reflected with culture‐dependent and ‐independent methods, which is generally acknowledged for that none of the current tools is able to disclose the whole picture of microbial diversity in environments (Lagier et al., 2012; Rettedal, Gumpert, & Sommer, 2014; Sommer, 2015).

**Figure 3:**
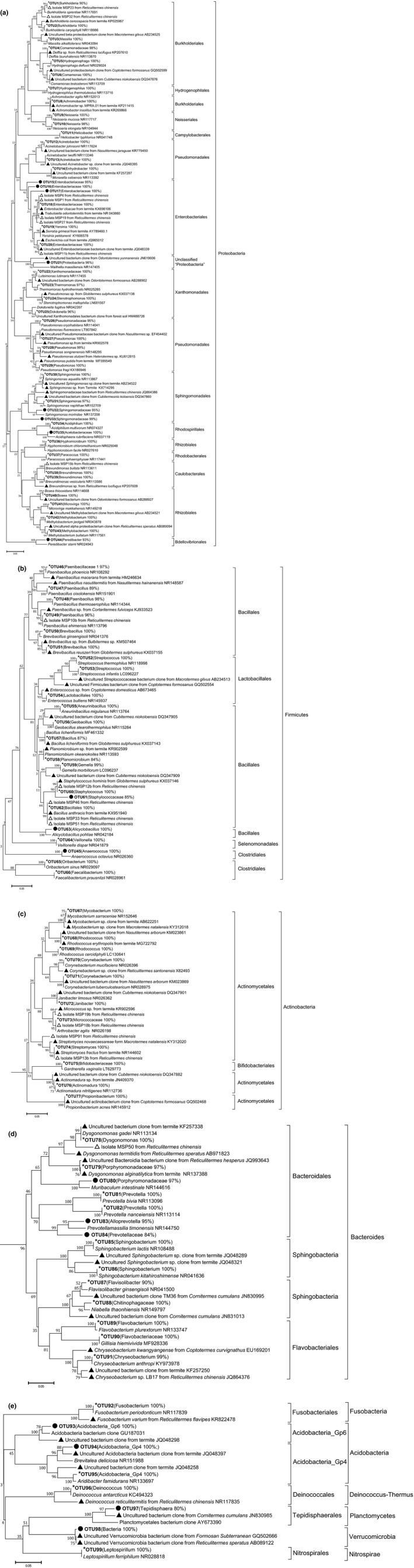
Phylogenetic trees of 99 OTUs from MSP pool. (a) *Proteobacteria*; (b) *Firmicutes*; (c) *Actinobacteria*; (d) *Bacteroides*; (e) OTUs belonging to other Phyla as indicated. The 16S rRNA sequences of all the published termite‐gut‐derived bacteria were mined from NCBI. The OTU sequences of MSP pool sample were blasted with the GenBank of NCBI and the 16S rRNA sequences of type species with the highest similarity to our OTUs were selected, together with previous mined termite gut derived bacterial 16s rRNA sequences, as reference sequences in phylogenetic tree. Tree viewing was inferred by the Neighbor‐joining method of Mega 6 based on the 16S rRNA gene sequences. OTUs obtained from this MSP pool were showed in bold. The most likely taxon category and its confidence value was listed in the bracket behind each OTU, the confidence threshold was set to be ≥80%. Symbols: ▲: Terminate sequence obtained from GenBank, accession numbers are shown at the end; ●: Sequence with 16S rRNA gene similarity lower than 97% compared with the other isolates in the environments; △: Strains isolated in this study, *: Cultivable taxa.

Phylogenetic trees were constructed based on the 99 OTUs from MSP pool (Figure 3a–e). Meanwhile, our data mining of public databases (Ribosomal Database Project, GreenGenes database, GenBank) revealed that 81 of the 99 OTUs (Figure 3, asterisk), representing 55 bacterial genera, had been well cultivated. But there were still 18 of the 99 OTUs, which had been previously not detected and not cultured (Figure 3, solid circle). The detection of these 18 OTUs in MSP pool indicated that they could grow in 1/5 R2A broth with MSP method. Indeed, we isolated and cultivated 396 bacterial strains with MSP method, and these strains covered 9.1% of the OTUs from MSP pool (Figure 3a–e). The identification of these bacterial strains is discussed in the following sections.

### 3.3 Isolation and cultivation of members from *Proteobacteria*

Analysis of the MSP pool data with RDP databank showed that there were 44 OTUs from *Proteobacteria* (Figure 3a). These 44 OTUs were assigned to 24 genera. We found that 12 of the 24 genera had been previously observed (Butera, Ferraro, Alonzo, Colazza, & Quatrini, 2016; Chou, Chen, Arun, & Young, 2007; Fall et al., 2007; Hongoh et al., 2006; Liu et al., 2013; Shinzato, Muramatsu, Matsui, & Watanabe, 2007; Thong‐On et al., 2012) but other 12 genera (*Massilia, Hydrogenophaga, Hydrogenophilus, Neisseria, Helicobacter, Thermomonas, Dokdonella, Acidiphilium, Hyphomicrobium, Paracoccus, Microvirga,* and *Peredibacter*) had not been reported previously for termite gut microbiota. We also found that there were eight of the 44 OTUs represented possible new taxa, as their 16S rRNA similarities to that of the currently known species were less than 97%. We obtained 351 isolates of *Proteobacteria*, from which four isolates belong to *Alphaproteobacteria*, 37 isolates belong to *Betaproteobacteria*, and 310 isolates belongs to *Gammaproteobacteria*. The members of family *Enterobacteriaceae* (307 isolates) and *Burkholderiaceae* (37 isolates) accounted for 98% (344/351) and they were the most abundant cultivable *Proteobacteria* in termite gut. As shown in Table 1, representative strains of the total isolates were good reflection of the MSP pool OTUs (See also Figure 3a), and two strains (MSP23, MSP32) that represented possible new species and/or genus were obtained.

**Table 1:** Bacterial strains isolated from the gut of *Reticulitermes chinensis* with MSP method

Phylogenetic affiliation	Strains	GenBank acc. No	Total isolates	Relatedness to known species
Proteobacteria				
* Brevundimonas*	MSP15	MG984086	4	*Brevundimonas terrae*, NR043726, 99%
* Burkholderia*	MSP23^a^	MG984075	25	*Burkholderia sacchari*, NR025097, 97%
	MSP32^a^	MG984074	12	*Burkholderia acidipaludis*, NR113024, 98%
* Luteibacter*	MSP17b	MG984089	2	*Luteibacter anthropi* NR116911, 99%
* Frateuria*	MSP1b	MG984088	1	*Frateuria aurantia* NR040947.1, 99%
* Escherichia*	MSP11b	MG984083	2	*Escherichia fergusonii* MF678858, 100%
* Citrobacter*	MSP27	MG984072	7	*Citrobacter farmeri* KT313001, 99%
* Trabulsiella*	MSP19	MG984073	16	*Trabulsiella odontotermitis* NR043860, 99%
* Enterobacter*	MSP1	MG984091	183	*Enterobacter amnigenus* DQ481471, 99%
	MSP6	MG984071	99	*Enterobacter amnigenus* DQ481471, 99%
Subtotal	10		351	
Firmicutes				
* Bacillus*	MSP33	MG984080	10	*Bacillus cereus*, KP694231, 100%
	MSP51	MG984081	10	*Bacillus thuringiensis*, EU647704, 99%
	MSP46	MG984082	6	*Bacillus wiedmannii*, MG780249, 100%
* Staphylococcus*	MSP12b	MG984084	2	*Staphylococcus epidermidis*, MG725753, 99%
* Lysinibacillus*	MSP8	MG984078	1	*Lysinibacillus xylanilyticus*, KY038731, 99%
	MSP58	MG984079	1	*Lysinibacillus macroides*, KF053268, 100%
	MSP14	MG984077	1	*Lysinibacillus macrolides*, KF053268, 99%
* Paenibacillus*	MSP10b	MG984070	1	*Paenibacillus ginsengagri*, AB245383, 99%
Subtotal	8		32	
Actinobacteria				
* Streptomyces*	MSP91	MG984076	4	*Streptomyces nigrogriseolus*, KF782837, 99%
	MSP13b	MG984085	3	*Streptomyces aureus*, NR025663, 99%
* Arthrobacter*	MSP18b	MG984087	1	*Arthrobacter cumminsii*, EU086827, 99%
* Micrococcus*	MSP19b^a^	MG984090	2	*Micrococcus luteus*, NR075062, 98%
Subtotal	4		10	
Bacteroidetes				
* Dysgonomonas*	MSP50^a^	MG984092	3	*Dysgonomonas gadei*, NR113134, 95%
Subtotal	1		3	
Total	23		396	

a Possible new species or genus.

Phylogenetic analysis also showed that most bacterial isolates were affiliated with several subgroups of *Proteobacteria* especially *Gammaproteobacteria*. *Enterobacter* strain MSP6 and MSP1 shared sequence similarity higher than 99% in 16S rRNA genes with those of the previously reported bacteria TSB7 and TSB75 (Fang et al., 2016) from *R. chinensis*. The *Citrobacter* strain MSP27 was closely related to the *Citrobacter* strains isolated from *R. chinensis* (Fang et al., 2016) and *R. speratus* (Cho, Kim, Kim, Kim, & Kim, 2010), whereas the *Trabulsiela* strain MSP19 was closely related to *Trabulsiella* strain LB10 (Fang et al., 2016) isolated from *R. chinensis* and *O. formosanus* (Chou et al., 2007). In addition, the 16S rRNA genes of strain MSP1b and MSP17b were similar to those of *Dyella* strains isolated from *R. chinensis* with sequence divergence less than 2%, and *Burkholderia* strains MSP23 and MSP32 were similar to strains TM6 and TSB14 from *R. chinensis* (Fang et al., 2016).

### 3.4 Isolation and cultivation of members from *Firmicutes*

*Firmicutes* was the secondly dominant phylum in the MSP pool of termite gut microbiota. Analysis of the MSP pool data with RDP databank showed that 21 OTUs were classified into *Firmicutes* (Figure 3b). These 21 OTUs were assigned to 14 genera. Closely related members (16S RNA similarity >97%) of the genera *Anaerococcus*,* Aneurinibacillus*,* Geobacillus*,* Gemella*,* Alicyclobacillus*,* Veillonella*,* Oribacterium,* and *Faecalibacterium* were the first time observed in termite gut microbiota. Totally 32 isolates were obtained with MSP method, and phylogenetic analysis showed that all the strains in the phylum *Firmicutes* were affiliated with four genera (*Bacillus*,* Lysinibacillus*,* Paenibacillus,* and *Staphylococcus*) (Table 1 and Figure 3b). Further 16S rRNA gene analysis showed that the majority of our strains shared high similarities to bacterial strains previously isolated from *R. chinensis* or other wood‐feeding termites. For example, strains MSP33, MSP46, MSP12b, and MSP10b shared 99% sequence similarities to members of *Bacillus*,* Staphaylococcus,* and *Paenibacillus* isolated from *R*. *chinensis* (Cibichakravarthy et al., 2017; Fang et al., 2016; Tarayre et al., 2013), whereas strains MSP14, MSP58, and MSP8 shared higher than 99% in 16S rRNA genes to members of *Lysinibacillus* isolated from *G. sulphureus* (Hussin & Majid, 2017).

### 3.5 Isolation and cultivation of members from *Actinobacteria*,* Bacteroidetes,* and other phyla

As showed in Table 1, there were 11 OTUs that corresponding to seven genera of *Actinobacteria* (Figure 3c), and 14 OTUs that corresponding to seven genera of *Bacteroidetes* (Figure 3d). Four strains were obtained (Table 1) and representing members of *Micrococcus* (MSP19b), *Arthrobacter* (MSP18b), and *Streptomyces* (MSP91, MSP13b). The 16S rRNA genes of strains MSP19b of *Actinobacteria* had similarities less than 98% to the previously reported microbial species, suggesting that it was possibly new species of *Micrococcus*. Compared to *Actinobacteria*, only three isolates belonging to *Bacteriodetes* (represented by strain MSP50) were obtained (Table 1), although more OTUs were recognized from the MSP pool (Figure 3d). Members of the genera *Prevotella, Alloprevotella, Flavisolibacter*, and *Flavobacterium* (Figure 3d) were the first time to be documented for termite gut microbiota. Figure 3d also shows that OTUs (such as OTU80, OTU83, OTU84, Similarity <97%) represented unclassified and possibly new taxa occurred in termite gut. For example, strain MSP50 shared only 95% of 16S rRNA gene sequence similarity to *Dysgonomonas gadi*, and represented a new member of *Bacteroidetes* and was closely associated with genus *Dysgonomonas*.

## 4 DISCUSSION

In this study, we continued our previous efforts to cultivate microbes from the termite gut (Chen et al., 2012; Fang et al., 2015, 2016) by application of the newly developed MSP method (Jiang et al., 2016). As a microfluidic technology (Ma et al., 2014; Tandogan et al., 2014), MSP method enables high‐throughput single‐cell cultivation of diverse bacterial groups and even rare species from environmental samples (Jiang et al., 2016). Comparing with other cultivation tools such as extinction‐culturing‐based method (Colin et al., 2013, 2015; Connon & Giovannoni, 2002), MSP technology has higher throughput as one culture‐plate can harbor thousands of droplets, whereas the extinction cultivation method carried only a few hundreds of wells. Other methods such as the microcapsulation and the Ichip (Keller & Zengler, 2004; Nichols et al., 2010) was reported to be applicable under exclusively aerobic condition, whereas the MSP approach can be used both aerobically and anaerobically. The MSP technique can be further exploited for extended applicability as (a) cocultivation of different microorganisms for the study of symbiotic interaction (Park et al., 2011), (b) recovering functional and rare biosphere members and (c) single‐cell sequencing.

We successfully cultivated a range of bacterial strains belonging to the *Delftia*,* Comamonas*,* Acinetobacter*,* Moraxella*,* Luteimona*,* Sphingomonas*,* Bosea*,* Methylobacterium*,* Corynebacterium*,* Janibacter*,* Propionibacterium,* and *Sphingobacterium* with MSP method in this study. Of note, the occurrence of these bacterial taxa in termite gut had been previously detected with molecular tools but they had not been cultivated (Butera et al., 2016; Diouf et al., 2015; Fall et al., 2007; Hongoh et al., 2005; Husseneder, Berestecky, & Grace, 2009; Matsui, Tanaka, Namihira, & Shinzato, 2012; Nakajima, Hongoh, Usami, Kudo, & Ohkuma, 2005; Thong‐On et al., 2012; Visser, Nobre, Currie, Aanen, & Poulsen, 2012; Zhu et al., 2012). The members of *Massilia*,* Hydrogenophaga*,* Hydrogenophilus*,* Neisseria*,* Helicobacter*,* Thermomonas*,* Dokdonella*,* Acidiphilium*,* Hyphomicrobium*,* Paracoccus*,* Microvirga*,* Peredibacter*,* Anaerococcus*,* Aneurinibacillus*,* Geobacillus*,* Gemella*,* Alicyclobacillus*,* Veillonella*,* Oribacterium*,* Faecalibacterium*,* Prevotella*,* Alloprevotella*,* Flavisolibacter,* and *Flavobacterium* were the first time to be documented for association with termite gut. Several possible new taxa were obtained with MSP method. The OTU21 represented an unclassified member of *Proteobacteria*, and its 16S RNA gene showed 90% similarity to *Mailhella massiliensis* (Ndongo et al., 2017). The OTU93 recovered in MSP pool represented a member of *Acidobacteria*, and its 16S RNA gene showed 94% similarity to *Vicinamibacter silvestris* (Huber et al., 2016); the OTU97 represented an unclassified member of *Phycisphaerae* and its closest relative is *Tepidisphaera mucosa* (Kovaleva et al., 2015) (Their 16S rRNA gene similarity is 91%); More interestingly, the OTU98 recovered from MSP pool in this study together with 16S rRNA gene sequences detected in Formosan subterranean termite (Husseneder et al., 2009) and *Reticulitermes speratus* (Hongoh, Ohkuma, & Kudo, 2003) clustered to a unique lineage of *Verrucomicrobia* (Figure 3e). So far, there is not any bacterial culture showing 16S rRNA gene sequence similarity higher than 80% to this unique lineage. Although we had not obtained pure cultures of those above OTUs, their occurrence in MSP pool indicated they did grow in MSP droplets. Further efforts to optimize their growth in droplets would result in finally obtaining their pure cultures.

Several strains that represent potential novel taxa were isolated with MSP method. *Dysgonomonas* (Hofstad et al., 2000) belongs to *Bacteroidetes*, and four species of *Dysgonomonas* were isolated from clinical specimen. Recently, two new species were isolated and characterized from termite guts, *Dysgonomonas macrotermitis* (Yang et al., 2014) and *Dysgonomonas termitidis* (Pramono, Sakamoto, Iino, Hongoh, & Ohkuma, 2015). In this study, we obtained three isolates, as represented by strain MSP50, and they are phylogenetically close to *Dysgonomonas*. The 16S rRNA gene of MSP50 showed 95% similarity to *Dysgonomonas gadei*. Whether MSP50 represents a novel species within *Dysgonomonas* or a novel genus within *Bacteroidetes* needs additional taxonomic studies.

There are many other bacterial taxa that existed in termite gut but have not been successfully cultivated in this study, such as the members of *Spirochaetes* and *Elusimicrobia* (see Supplementary material Table S1). *Spirochaetes* widely occur in wood‐feeding termites and are the most abundant bacterial symbionts in *Reticulitermes* (Brune & Dietrich, 2015; Graber & Breznak, 2005; Noda, Ohkuma, Yamada, Hongoh, & Kudo, 2003). *Elusimicrobia* are found almost exclusively in the intestinal tract of animals and are particularly abundant in lower termites, where they reside as intracellular symbionts in the cellulolytic gut flagellates (Geissinger, Herlemann, Mörschel, Maier, & Brune, 2009; Ikeda‐Ohtsubo, Faivre, & Brune, 2010; Zheng, Dietrich, Radek, & Brune, 2016). The rarefaction curves of observed taxa in Figure 1a indicated that the achieved isolates can only recover a fraction of total species in termite gut. However, the observed recovery rate might be further improved by utilization of various cultural media and different culture conditions. Specifically, further efforts should be made to apply low but diverse nutrients in culture broth, besides 1/5 R2A, at various aerobic and anoxic levels for harvest of additional bacterial groups that are fastidious to nutrients and sensitive to oxidoreductive states.

In this study, 99 OTUs were identified to be cultivable with culture‐dependent MSP method, whereas 353 OTUs were detected from the termite gut microbiota sample (OMG) using the culture‐independent metagenomic method. This result showed that, only a fraction of the cultivable taxa (24 of 99 OTUs) was detectable with metagenomic method. Similar observation was reported by Lagier et al. (2012). In their study, 340 bacterial species were cultured from human gut using the MALDI‐TOF‐based culturomic strategy, and only 15% (51 species) of these cultivable isolates were detected by metagenomic pyrosequencing. The results manifested that culturomics complemented metagenomic by overcoming the depth bias inherent in metagenomic approaches (Lagier et al., 2012). Later on, Rettedal et al. (2014) demonstrated that the recovery rate and representativeness of culture‐dependent approaches in gut microbiota could be further improved by careful design of culture conditions. It is believed that, by optimization of the culture conditions, MSP method would have better performance in microbiome recovery.

## References

[mbo3654-bib-0001] Ali, S. S. , Wu, J. , Xie, R. , Zhou, F. , Sun, J. , & Huang, M. (2017). Screening and characterizing of xylanolytic and xylose‐fermenting yeasts isolated from the wood‐feeding termite, *Reticulitermes chinensis* . PLoS ONE, 12(7), e0181141 10.1371/journal.pone.0181141 28704553 PMC5509302

[mbo3654-bib-0002] Amann, R. I. , Ludwig, W. , & Schleifer, K. H. (1995). Phylogenetic identification of individual microbial cells without cultivation. Microbiological Reviews, 59(1), 143–169. 7535888 10.1128/mr.59.1.143-169.1995 PMC239358

[mbo3654-bib-0003] Auer, L. , Lazuka, A. , Sillamdussès, D. , Miambi, E. , O'Donohue, M. , & Hernandezraquet, G. (2017). Uncovering the potential of termite gut microbiome for lignocellulose bioconversion in anaerobic batch bioreactors. Frontiers in Microbiology, 8, 2623 10.3389/fmicb.2017.02623 29312279 PMC5744482

[mbo3654-bib-0004] Bignell, D. E. (2011). Morphology, physiology, biochemistry and functional design of the termite gut: An evolutionary wonderland In Bignell D. E., Roisin Y., & Lo N. (Eds.), Biology of termites: A modern synthesis (pp. 375–412). Netherland: Springer 10.1007/978-90-481-3977-4

[mbo3654-bib-0005] Bokulich, N. A. , Subramanian, S. , Faith, J. J. , Gevers, D. , Gordon, J. I. , Knight, R. , … Caporaso, J. G. (2013). Quality‐filtering vastly improves diversity estimates from illumina amplicon sequencing. Nature Methods, 10(1), 57 10.1038/nmeth.2276 23202435 PMC3531572

[mbo3654-bib-0006] Breznak, J. A. (1982). Intestinal microbiota of termites and other xylophagous insects. Annual Review of Microbiology, 36(1), 323–343. 10.1146/annurev.mi.36.100182.001543 6756291

[mbo3654-bib-0007] Breznak, J. A. (2000). Ecology of prokaryotic microbes in the guts of wood‐ and litter‐feeding termites In Abe Y., Bignell D. E., & Higashi T. (Eds.), Termites: Evolution, Sociality, Symbioses, Ecology (pp. 209–231). Netherlands: Kluwer Academic Publishers 10.1007/978-94-017-3223-9

[mbo3654-bib-0008] Brune, A. , & Dietrich, C. (2015). The gut microbiota of termites: Digesting the diversity in the light of ecology and evolution. Annual Review of Microbiology, 69(1), 145 10.1146/annurev-micro-092412-155715 26195303

[mbo3654-bib-0009] Brune, A. , & Friedrich, M. (2000). Microecology of the termite gut: Structure and function on a microscale. Current Opinion in Microbiology, 3(3), 263–269. 10.1016/S1369-5274(00)00087-4 10851155

[mbo3654-bib-0010] Butera, G. , Ferraro, C. , Alonzo, G. , Colazza, S. , & Quatrini, P. (2016). The gut microbiota of the wood‐feeding termite *Reticulitermes lucifugus* (isoptera; Rhinotermitidae). Annals of Microbiology, 66(1), 253–260. 10.1007/s13213-015-1101-6

[mbo3654-bib-0011] Chen, W. , Wang, B. , Hong, H. , Yang, H. , & Liu, S. J. (2012). *Deinococcus reticulitermitis* sp. nov., isolated from a termite gut. International Journal of Systematic & Evolutionary Microbiology, 62(1), 78–83. 10.1099/ijs.0.026567-0 21335505

[mbo3654-bib-0012] Cho, M. J. , Kim, Y. K. , Kim, Y. K. , Kim, Y. S. , & Kim, T. J. (2010). Symbiotic adaptation of bacteria in the gut of *Reticulitermes speratus*: Low endo‐beta‐1,4‐glucanase activity. Biochemical & Biophysical Research Communications, 395(3), 432–435. 10.1016/j.bbrc.2010.04.048 20385103

[mbo3654-bib-0013] Chou, J. H. , Chen, W. M. , Arun, A. B. , & Young, C. C. (2007). *Trabulsiella odontotermitis* sp. nov., isolated from the gut of the termite *Odontotermes formosanus* shiraki. International Journal of Systematic & Evolutionary Microbiology, 57(4), 696–700. 10.1099/ijs.0.64632-0 17392189

[mbo3654-bib-0014] Cibichakravarthy, B. , Abinaya, S. , & Prabagaran, S. R. (2017). Syntrophic association of termite gut bacterial symbionts with bifunctional characteristics of cellulose degrading and polyhydroxyalkanoate producing bacteria. International Journal of Biological Macromolecules, 103, 613–620. 10.1016/j.ijbiomac.2017.05.100 28528947

[mbo3654-bib-0015] Colin, Y. , Goñiurriza, M. , Caumette, P. , & Guyoneaud, R. (2013). Combination of high throughput cultivation and dsrA sequencing for assessment of sulfate‐reducing bacteria diversity in sediments. FEMS Microbiology Ecology, 83(1), 26–37. 10.1111/j.1574-6941.2012.01452.x 22809466

[mbo3654-bib-0016] Colin, Y. , Goñi‐Urriza, M. , Caumette, P. , & Guyoneaud, R. (2015). Contribution of enrichments and resampling for sulfate reducing bacteria diversity assessment by high‐throughput cultivation. Journal of Microbiol Methods, 110, 92–97. 10.1016/j.mimet.2015.01.003 25578508

[mbo3654-bib-0017] Connon, S. A. , & Giovannoni, S. J. (2002). High‐throughput methods for culturing microorganisms in very‐low‐nutrient media yield diverse new marine isolates. Applied & Environmental Microbiology, 68(8), 3878–3885. 10.3410/f.1008856.113558 12147485 PMC124033

[mbo3654-bib-0018] Desantis, T. Z. , Hugenholtz, P. , Larsen, N. , Rojas, M. , Brodie, E. L. , Keller, K. , … Andersen, G. L. (2006). Greengenes, a chimera‐checked 16S rRNA gene database and workbench compatible with ARB. Applied & Environmental Microbiology, 72(7), 5069–5072. 10.1128/AEM.03006-05 16820507 PMC1489311

[mbo3654-bib-0019] Diouf, M. , Roy, V. , Mora, P. , Frechault, S. , Lefebvre, T. , Hervé, V. , … Miambi, E. (2015). Profiling the succession of bacterial communities throughout the life stages of a higher termite *Nasutitermes arborum* (Termitidae, Nasutitermitinae) using 16S rRNA gene pyrosequencing. PLoS ONE, 10(10), e0140014 10.1371/journal.pone.0140014 26444989 PMC4596844

[mbo3654-bib-0020] Edgar, R. C. (2013). UPARSE: Highly accurate OTU sequences from microbial amplicon reads. Nature Methods, 10(10), 996–998. 10.1038/NMETH.2604 23955772

[mbo3654-bib-0021] Edwards, U. , Rogall, T. , Blöcker, H. , Emde, M. , & Böttger, A. E. C. (1989). Isolation and direct complete nucleotide determination of entire genes. Characterization of a gene coding for 16s ribosomal RNA. Nucleic Acids Research, 17(19), 7843–7853. 10.1093/nar/17.19.7843 2798131 PMC334891

[mbo3654-bib-0022] Fall, S. , Hamelin, J. , Ndiaye, F. , Assigbetse, K. , Aragno, M. , Chotte, J. L. , & Brauman, A. (2007). Differences between bacterial communities in the gut of a soil‐feeding termite (*Cubitermes niokoloensis*) and its mounds. Applied & Environmental Microbiology, 73(16), 5199–5208. 10.1128/AEM.02616-06 17574999 PMC1950997

[mbo3654-bib-0023] Fang, H. , Chen, W. , Wang, B. J. , Li, X. J. , Liu, S. J. , & Yang, H. (2016). Cultivation and characterization of symbiotic bacteria from the gut of *Reticulitermes chinensis* . Applied Environmental Biotechnology, 1(1), 3–12. 10.18063/AEB.2016.01.004

[mbo3654-bib-0024] Fang, H. , Lv, W. , Huang, Z. , Liu, S. J. , & Yang, H. (2015). *Gryllotalpicola reticulitermitis* sp. nov., isolated from a termite gut. International Journal of Systematic & Evolutionary Microbiology, 65(1), 85–89. 10.1099/ijs.0.062984-0 25281726

[mbo3654-bib-0025] Geissinger, O. , Herlemann, D. P. , Mörschel, E. , Maier, U. G. , & Brune, A. (2009). The ultramicrobacterium “*Elusimicrobium minutum*” gen. nov., sp. nov., the first cultivated representative of the termite group 1 phylum. Applied & Environmental Microbiology, 75(9), 2831–2840. 10.1128/AEM.02697-08 19270135 PMC2681718

[mbo3654-bib-0026] Graber, J. R. , & Breznak, J. A. (2005). Folate cross‐feeding supports symbiotic homoacetogenic spirochetes. Applied & Environmental Microbiology, 71(4), 1883–1889. 10.1128/AEM.71.4.1883-1889.2005 15812016 PMC1082566

[mbo3654-bib-0027] Hofstad, T. , Olsen, I. , Eribe, E. R. , Falsen, E. , Collins, M. D. , & Lawson, P. A. (2000). *Dysgonomonas* gen. nov. to accommodate *Dysgonomonas gadei* sp. nov., an organism isolated from a human gall bladder, and *Dysgonomonas capnocytophagoides* (formerly CDC group DF‐3). International Journal of Systematic & Evolutionary Microbiology, 50(6), 2189–2195. 10.1099/00207713-50-6-2189 11155996

[mbo3654-bib-0028] Hongoh, Y. (2011). Toward the functional analysis of uncultivable, symbiotic microorganisms in the termite gut. Cellular and Molecular Life Sciences: CMLS, 68(8), 1311–1325. 10.1007/s00018-011-0648-z 21365277 PMC11114660

[mbo3654-bib-0029] Hongoh, Y. , Deevong, P. , Inoue, T. , Moriya, S. , Trakulnaleamsai, S. , Ohkuma, M. , … Kudo, T. (2005). Intra‐ and interspecific comparisons of bacterial diversity and community structure support coevolution of gut microbiota and termite host. Applied & Environmental Microbiology, 71(11), 6590–6599. 10.1128/AEM.71.11.6590-6599.2005 16269686 PMC1287746

[mbo3654-bib-0030] Hongoh, Y. , Ekpornprasit, L. , Inoue, T. , Moriya, S. , Trakulnaleamsai, S. , Ohkuma, M. , … Kudo, T. (2006). Intracolony variation of bacterial gut microbiota among castes and ages in the fungus‐growing termite *Macrotermes gilvus* . Molecular Ecology, 15(2), 505–516. 10.1111/j.1365-294X.2005.02795.x 16448416

[mbo3654-bib-0031] Hongoh, Y. , Ohkuma, M. , & Kudo, T. (2003). Molecular analysis of bacterial microbiota in the gut of the termite *Reticulitermes speratus* (Isoptera; Rhinotermitidae). FEMS Microbiology Ecology, 44(2), 231–242. 10.1016/S0168-6496(03)00026-6 19719640

[mbo3654-bib-0032] Huang, X. F. , Bakker, M. G. , Judd, T. M. , Reardon, K. F. , & Vivanco, J. M. (2013). Variations in diversity and richness of gut bacterial communities of termites (*Reticulitermes flavipes*) fed with grassy and woody plant substrates. Microbial Ecology, 65(3), 531–536. 10.1007/s00248-013-0219-y 23529653

[mbo3654-bib-0033] Huber, K. J. , Geppert, A. M. , Wanner, G. , Fösel, B. U. , Wüst, P. K. , & Overmann, J. (2016). The first representative of the globally widespread subdivision 6 Acidobacteria, *Vicinamibacter silvestris* gen. nov., sp. nov., isolated from subtropical savannah soil. International Journal of Systematic & Evolutionary Microbiology, 66(8), 2971–2979. 10.1099/ijsem.0.001131 27150379

[mbo3654-bib-0034] Husseneder, C. , Berestecky, J. M. , & Grace, J. K. (2009). Changes in composition of culturable bacteria community in the gut of the *Formosan subterranean* termite depending on rearing conditions of the host. Annals of the Entomological Society of America, 102(3), 498–507. 10.1603/008.102.0321

[mbo3654-bib-0035] Hussin, N. A. , & Majid, A. H. A. (2017). Inter and intra termites colonies comparisons of gut microbial diversity from worker and soldier caste of *Globitermes sulphureus* (Blattodea: Termitidae) using 16S rRNA gene. Malaysian Journal of Microbiology, 13(3), 228–234.

[mbo3654-bib-0036] Ikeda‐Ohtsubo, W. , Faivre, N. , & Brune, A. (2010). Putatively free‐living ‘Endomicrobia’‐ ancestors of the intracellular symbionts of termite gut flagellates? Environmental Microbiology Reports, 2(4), 554–559. 10.1111/j.1758-2229.2009.00124.x 23766225

[mbo3654-bib-0037] Jiang, C. Y. , Dong, L. , Zhao, J. K. , Hu, X. , Shen, C. , Qiao, Y. , … Du, W. (2016). High‐throughput single‐cell cultivation on microfluidic streak plates. Applied & Environmental Microbiology, 82(7), 2210–2218. 10.1128/AEM.03588-15 26850294 PMC4807504

[mbo3654-bib-0038] Keller, M. , & Zengler, K. (2004). Tapping into microbial diversity. Nature Reviews Microbiology, 2(2), 141–150. 10.1038/nrmicro819 15040261

[mbo3654-bib-0039] Kovaleva, O. L. , Merkel, A. Y. , Novikov, A. A. , Baslerov, R. V. , Toshchakov, S. V. , & Bonch‐Osmolovskaya, E. A. (2015). *Tepidisphaera mucosa* gen. nov., sp. nov., a moderately thermophilic member of the class Phycisphaerae in the phylum Planctomycetes, and proposal of a new family, Tepidisphaeraceae fam. nov., and a new order, Tepidisphaerales ord. nov. International Journal of Systematic & Evolutionary Microbiology, 65(2), 549–555. 10.1099/ijs.0.070151-0 25404483

[mbo3654-bib-0040] Lagier, J. C. , Armougom, F. , Million, M. , Hugon, P. , Pagnier, I. , Robert, C. , … Raoult, D. (2012). Microbial culturomics: Paradigm shift in the human gut microbiome study. Clinical Microbiology & Infection, 18(12), 1185–1193. 10.1111/1469-0691.12023 23033984

[mbo3654-bib-0041] Liu, N. , Yan, X. , Zhang, M. , Xie, L. , Wang, Q. , Huang, Y. , … Zhou, Z. (2011). Microbiome of fungus‐growing termites: A new reservoir for lignocellulase genes. Applied & Environmental Microbiology, 77(1), 48–56. 10.1128/AEM.01521-10 21057022 PMC3019695

[mbo3654-bib-0042] Liu, N. , Zhang, L. , Zhou, H. , Zhang, M. , Yan, X. , Wang, Q. , … Zhou, Z. (2013). Metagenomic insights into metabolic capacities of the gut microbiota in a fungus‐cultivating termite (*Odontotermes yunnanensis*). PLoS ONE, 8(7), e69184 10.1371/journal.pone.0069184 23874908 PMC3714238

[mbo3654-bib-0043] Ma, L. , Kim, J. , Hatzenpichler, R. , Karymov, M. A. , Hubert, N. , Hanan, I. M. , … Ismagilov, R. F. (2014). Gene‐targeted microfluidic cultivation validated by isolation of a gut bacterium listed in Human Microbiome Project's Most Wanted taxa. Proceedings of the National Academy of Sciences of the United States of America, 111(27), 9768–9773. 10.1073/pnas.1404753111 24965364 PMC4103313

[mbo3654-bib-0044] Magoč, T. , & Salzberg, S. L. (2011). FLASH: Fast length adjustment of short reads to improve genome assemblies. Bioinformatics, 27(21), 2957–2963. 10.1093/bioinformatics/btr507 21903629 PMC3198573

[mbo3654-bib-0045] Martin, M. M. , & Martin, J. S. (1978). Cellulose digestion in the midgut of the fungus‐growing termite *Macrotermes natalensis*: The role of acquired digestive enzymes. Science, 199(4336), 1453–1455. 10.1126/science.199.4336.1453 17796679

[mbo3654-bib-0046] Matsui, T. , Tanaka, J. , Namihira, T. , & Shinzato, N. (2012). Antibiotics production by an Actinomycete isolated from the termite gut. Journal of Basic Microbiology, 52(6), 731–735. 10.1002/jobm.201100500 22359219

[mbo3654-bib-0047] Matsuura, K. , Yashiro, T. , Shimizu, K. , Tatsumi, S. , & Tamura, T. (2009). Cuckoo fungus mimics termite eggs by producing the cellulose‐digesting enzyme β‐glucosidase. Current Biology, 19(1), 30–36. 10.1016/j.cub.2008.11.030 19110429

[mbo3654-bib-0048] Nakajima, H. , Hongoh, Y. , Usami, R. , Kudo, T. , & Ohkuma, M. (2005). Spatial distribution of bacterial phylotypes in the gut of the termite *Reticulitermes speratus* and the bacterial community colonizing the gut epithelium. FEMS Microbiology Ecology, 54(2), 247–255. 10.1016/j.femsec.2005.03.010 16332323

[mbo3654-bib-0049] Ndongo, S. , Cadoret, F. , Dubourg, G. , Delerce, J. , Fournier, P. E. , Raoult, D. , & Lagier, J. C. (2017). ‘*Collinsella phocaeensis*’ sp. nov., ‘*Clostridium merdae*’ sp. nov., ‘*Sutterella massiliensis*’ sp. nov., ‘*Sutturella timonensis*’ sp. nov., ‘*Enorma phocaeensis*’ sp. nov., ‘*Mailhella massiliensis*’ gen. nov., sp. nov., ‘*Mordavella massiliensis*’ gen. nov., sp. nov. and ‘*Massiliprevotella massiliensis*’ gen. nov., sp. nov., 9 new species isolated from fresh stool samples of healthy French patients. New Microbes and New Infections, 17, 89–95. 10.1016/j.nmni.2017.02.005 28409003 PMC5382032

[mbo3654-bib-0050] Ni, J. , & Tokuda, G. (2013). Lignocellulose‐degrading enzymes from termites and their symbiotic microbiota. Biotechnology Advances, 31(6), 838–850. 10.1016/j.biotechadv.2013.04.005 23623853

[mbo3654-bib-0051] Nichols, D. , Cahoon, N. , Trakhtenberg, E. M. , Pham, L. , Mehta, A. , Belanger, A. , … Epstein, S. S. (2010). Use of ichip for high‐throughput in situ cultivation of “uncultivable” microbial species. Applied & Environmental Microbiology, 76(8), 2445–2450. 10.1128/aem.01754-09 20173072 PMC2849220

[mbo3654-bib-0052] Noda, S. , Ohkuma, M. , Yamada, A. , Hongoh, Y. , & Kudo, T. (2003). Phylogenetic position and in situ identification of ectosymbiotic spirochetes on protists in the termite gut. Applied & Environmental Microbiology, 69(1), 625–633. 10.1128/AEM.69.1.625-633.2003 12514050 PMC152436

[mbo3654-bib-0053] Ohkuma, M. (2003). Termite symbiotic systems: Efficient bio‐recycling of lignocellulose. Applied Microbiology & Biotechnology, 61(1), 1–9. 10.1371/10.1007/s00253-002-1189-z 12658509

[mbo3654-bib-0054] Ohkuma, M. , & Brune, A. (2011). Diversity, structure, and evolution of the termite gut microbial community In Bignell D. E., Roisin Y., & Lo N. (Eds.), Biology of termites: A modern synthesis (pp. 413–438). Netherlands: Springer.

[mbo3654-bib-0055] Ohkuma, M. , & Kudo, T. (1996). Phylogenetic diversity of the intestinal bacterial community in the termite *Reticulitermes speratus* . Applied & Environmental Microbiology, 62(2), 461–468. 10.1128/JB.00345-12 8593049 PMC167814

[mbo3654-bib-0056] Park, J. , Kerner, A. , Burns, M. A. , & Lin, X. N. (2011). Microdroplet‐enabled highly parallel co‐cultivation of microbial communities. PLoS ONE, 6(2), e17019 10.1371/journal.pone.0017019 21364881 PMC3045426

[mbo3654-bib-0057] Pramono, A. K. , Sakamoto, M. , Iino, T. , Hongoh, Y. , & Ohkuma, M. (2015). *Dysgonomonas termitidis* sp. nov., isolated from the gut of the subterranean termite *Reticulitermes speratus* . International Journal of Systematic & Evolutionary Microbiology, 65(2), 681–685. 10.1099/ijs.0.070391-0 25428419

[mbo3654-bib-0058] Rettedal, E. A. , Gumpert, H. , & Sommer, M. O. (2014). Cultivation‐based multiplex phenotyping of human gut microbiota allows targeted recovery of previously uncultured bacteria. Nature Communications, 5, 4714 10.1038/ncomms5714 25163406

[mbo3654-bib-0059] Shinzato, N. , Muramatsu, M. , Matsui, T. , & Watanabe, Y. (2007). Phylogenetic analysis of the gut bacterial microflora of the fungus‐growing termite *Odontotermes formosanus* . Bioscience, Biotechnology, and Biochemistry, 71(4), 906–915. 10.1271/bbb.60540 17420599

[mbo3654-bib-0060] Sommer, M. O. (2015). Advancing gut microbiome research using cultivation. Current Opinion in Microbiology, 27, 127–132. 10.1016/j.mib.2015.08.004 26401902

[mbo3654-bib-0061] Stewart, E. J. (2012). Growing unculturable bacteria. Journal of Bacteriology, 194(16), 4151–4160. 10.1128/JB.00345-12 22661685 PMC3416243

[mbo3654-bib-0062] Tandogan, N. , Abadian, P. N. , Epstein, S. , Aoi, Y. , & Goluch, E. D. (2014). Isolation of microorganisms using sub‐micrometer constrictions. PLoS ONE, 9(6), e101429 10.1371/journal.pone.0101429 24978477 PMC4076310

[mbo3654-bib-0063] Tarayre, C. , Bauwens, J. , Mattéotti, C. , Brasseur, C. , Millet, C. , Massart, S. , … Delvigne, F. (2015). Multiple analyses of microbial communities applied to the gut of the wood‐feeding termite *Reticulitermes flavipes,* fed on artificial diets. Symbiosis, 65(3), 143–155. 10.1007/s13199-015-0328-0

[mbo3654-bib-0064] Tarayre, C. , Brognaux, A. , Brasseur, C. , Bauwens, J. , Millet, C. , Mattéotti, C. , … Thonart, P. (2013). Isolation and cultivation of a xylanolytic *Bacillus subtilis* extracted from the gut of the termite *Reticulitermes santonensis* . Applied Biochemistry & Biotechnology, 171(1), 225–245. 10.1007/s12010-013-0337-5 23828225

[mbo3654-bib-0065] Thompson, J. D. , Gibson, T. J. , & Higgins, D. G. (2002). Multiple sequence alignment using ClustalW and ClustalX. Current Protocols in Bioinformatics, 2, Unit 2.3 10.1002/0471250953.bi0203s00 18792934

[mbo3654-bib-0066] Thong‐On, A. , Suzuki, K. , Noda, S. , Inoue, J. I. , Kajiwara, S. , & Ohkuma, M. (2012). Isolation and characterization of anaerobic bacteria for symbiotic recycling of uric acid nitrogen in the gut of various termites. Microbes and Environments, 27(2), 186–192. 10.1264/jsme2.ME11325 22791052 PMC4036019

[mbo3654-bib-0067] Visser, A. A. , Nobre, T. , Currie, C. R. , Aanen, D. K. , & Poulsen, M. (2012). Exploring the potential for actinobacteria as defensive symbionts in fungus‐growing termites. Microbial Ecology, 63(4), 975–985. 10.1007/s00248-011-9987-4 22173371

[mbo3654-bib-0068] Wang, Q. , Garrity, G. M. , Tiedje, J. M. , & Cole, J. R. (2007). Naive Bayesian classifier for rapid assignment of rRNA sequences into the new bacterial taxonomy. Applied & Environmental Microbiology, 73(16), 5261–5267. 10.1128/AEM.00062-07 17586664 PMC1950982

[mbo3654-bib-0069] Warnecke, F. , Luginbühl, P. , Ivanova, N. , Ghassemian, M. , Richardson, T. H. , Stege, J. T. , … Leadbetter, J. R. (2007). Metagenomic and functional analysis of hindgut microbiota of a wood‐feeding higher termite. Nature, 450(7169), 560–565. 10.1038/nature06269 18033299

[mbo3654-bib-0070] Watanabe, H. , & Tokuda, G. (2010). Cellulolytic systems in insects. Annual Reviews of Entomology, 55, 609–632. 10.1146/annurev-ento-112408-085319 19754245

[mbo3654-bib-0071] Weisburg, W. G. , Bars, S. M. , Pelletier, D. A. , & Lane, D. J. (1991). 16S Ribosomal DNA amplification for phylogenetic study. Journal of Bacteriology, 173(2), 697–703. 10.1128/jb.173.2.697-703.1991 1987160 PMC207061

[mbo3654-bib-0072] Werner, J. J. , Zhou, D. , Caporaso, J. G. , Knight, R. , & Angenent, L. T. (2012). Comparison of Illumina paired‐end and single‐direction sequencing for microbial 16S rRNA gene amplicon surveys. The ISME Journal, 6(7), 1273–1276. 10.1038/ismej.2011.186 22170427 PMC3379627

[mbo3654-bib-0073] Yang, Y. J. , Zhang, N. , Ji, S. Q. , Lan, X. , Zhang, K. D. , Shen, Y. L. , … Ni, J. F. (2014). *Dysgonomonas macrotermitis* sp. nov., isolated from the hindgut of a fungus‐growing termite. International Journal of Systematic & Evolutionary Microbiology, 64(9), 2956–2961. 10.1099/ijs.0.061739-0 24899656

[mbo3654-bib-0074] Zheng, H. , Dietrich, C. , Radek, R. , & Brune, A. (2016). *Endomicrobium proavitum*, the first isolate of Endomicrobia class. nov. (phylum Elusimicrobia)–an ultramicrobacterium with an unusual cell cycle that fixes nitrogen with a Group IV nitrogenase. Environmental Microbiology, 18(1), 191–204. 10.1111/1462-2920.12960 26119974

[mbo3654-bib-0075] Zhou, Q. Z. , Liu, X. Y. , Liu, S. J. , Ma, G. H. , & Su, Z. G. (2008). Preparation of uniformly sized agarose microcapsules by membrane emulsification for application in sorting bacteria. Industrial & Engineering Chemistry Research, 47(17), 6386–6390. 10.1021/ie800011r

[mbo3654-bib-0076] Zhu, Y. H. , Li, J. , Liu, H. H. , Yang, H. , Xin, S. , Zhao, F. , … Lu, X. Y. (2012). Phylogenetic analysis of the gut bacterial microflora of the fungus‐growing termite macrotermes barneyi. African Journal of Microbiology Research, 6(9), 2071–2078. 10.5897/AJMR11.1345

