# Supplementary material for: Harnessing microfluidic streak plate technique to investigate the gut microbiome of Reticulitermes chinensis
Source: Microbiologyopen. 2018 Jun 13;8(3):e00654. doi: 10.1002/mbo3.654 (PMC6436436; doi:10.1002/mbo3.654)
Supplement: Supplementary file 1 [file MBO3-8-e00654-s001.docx]

Supplementary materials to:

**Harnessing the gut microbiome of *Reticulitermes chinensis* with microdroplet streaking plate technique**

**Nan Zhou^1,2^, Yu-Tong Sun^1,3^, Dong-Wei Chen^1^, Wenbin Du^1^, Hong Yang^4^, and Shuang-Jiang Liu^1,2,3^**

^1^State Key Laboratory of Microbial Resources and Environmental Microbiology Research Center at Institute of Microbiology, Chinese Academy of Sciences, Beijing 100101, China; ^2^University of Chinese Academy of Sciences, 100101 Beijing, China; ^3^College of Life Science at Hebei University, Baoding, China; ^4^School of Life Sciences at Central China Normal University, Wuhan, 430079, China

***Corresponding to:**

**Hong Yang:** [**hyang@mail.ccnu.edu.cn**](mailto:hyang@mail.ccnu.edu.cn)

**Shuang-Jiang Liu: liusj@im.ac.cn**

Supplementary materials

Table S1. Microbial community compositions at phylum level

| Taxon | MSP (%) | OMG(%) |
| --- | --- | --- |
| *Acidobacteria* | 0.052976 | 0 |
| *Actinobacteria* | 0.386213 | 0.848152 |
| *Bacteroidetes* | 0.317856 | 9.960443 |
| *Elusimicrobia* | 0 | 13.78782 |
| *Firmicutes* | 29.16759 | 13.86266 |
| *Fusobacteria* | 0.059812 | 0 |
| *Nitrospirae* | 0.011962 | 0 |
| *Planctomycetes* | 0.003418 | 0.270839 |
| *Proteobacteria* | 69.9489 | 14.73932 |
| *Spirochaetes* | 0 | 44.32843 |
| *Synergistetes* | 0 | 0.516731 |
| *Tenericutes* | 0 | 0.972881 |
| *Thermi* | 0.008545 | 0 |
| *Verrucomicrobia* | 0.042723 | 0.580877 |
| ZB3 | 0 | 0.131856 |

Table S2. Community profiling based on metagenomic sequencing of bacterial 16S rRNA genes. OTU abundances were given for pooled cells from MSPs

| OTU/N | Taxon | relative abundance |
| --- | --- | --- |
| OTU1 | Proteobacteria;Betaproteobacteria;Burkholderiales;Burkholderiaceae;Burkholderia | 0.765759606 |
| OTU2 | Proteobacteria;Betaproteobacteria;Burkholderiales;Burkholderiaceae;Burkholderia | 0.032476412 |
| OTU3 | Proteobacteria;Betaproteobacteria;Burkholderiales;Oxalobacteraceae;Massilia | 0.023929988 |
| OTU4 | Proteobacteria;Betaproteobacteria;Burkholderiales;Comamonadaceae;Delftia | 0.046150691 |
| OTU5 | Proteobacteria;Betaproteobacteria;Burkholderiales;Comamonadaceae;Hydrogenophaga | 0.018802133 |
| OTU6 | Proteobacteria;Betaproteobacteria;Burkholderiales;Comamonadaceae;Comamonas | 0.458088336 |
| OTU7 | Proteobacteria;Betaproteobacteria;Rhodocyclales;Rhodocyclaceae;Hydrogenophilus | 0.010255709 |
| OTU8 | Proteobacteria;Betaproteobacteria;Burkholderiales;Alcaligenaceae;Achromobacter | 9.683098592 |
| OTU9 | Proteobacteria;Betaproteobacteria;Neisseriales;Neisseriaceae;Neisseria | 0.044441406 |
| OTU10 | Proteobacteria;Betaproteobacteria;Neisseriales;Neisseriaceae;Neisseria | 0.022220703 |
| OTU11 | Proteobacteria;Epsilonproteobacteria;Campylobacterales;Helicobacteraceae;Helicobacter | 0.001709285 |
| OTU12 | Proteobacteria;Gammaproteobacteria;Pseudomonadales;Moraxellaceae;Acinetobacter | 0.203404895 |
| OTU13 | Proteobacteria;Gammaproteobacteria;Pseudomonadales;Moraxellaceae;Acinetobacter | 0.420484069 |
| OTU14 | Proteobacteria;Gammaproteobacteria;Pseudomonadales;Moraxellaceae;Enhydrobacter | 0.299124846 |
| OTU15 | Proteobacteria;Gammaproteobacteria;Enterobacteriales;Enterobacteriaceae | 0.020511418 |
| OTU16 | Proteobacteria;Gammaproteobacteria;Enterobacteriales;Enterobacteriaceae | 0.976001641 |
| OTU17 | Proteobacteria;Gammaproteobacteria;Enterobacteriales;Enterobacteriaceae | 0.00341857 |
| OTU18 | Proteobacteria;Gammaproteobacteria;Enterobacteriales;Enterobacteriaceae | 9.951456311 |
| OTU19 | Proteobacteria;Gammaproteobacteria;Enterobacteriales;Enterobacteriaceae;Yersinia | 1.546902776 |
| OTU20 | Proteobacteria;Gammaproteobacteria;Enterobacteriales;Enterobacteriaceae; Escherichia/Shigella | 21.10624915 |
| OTU21 | Proteobacteria;Deltaproteobacteria;Desulfovibrionales;Desulfovibrionaceae | 0.010255709 |
| OTU22 | Proteobacteria;Gammaproteobacteria;Xanthomonadales;Xanthomonadaceae; Luteimonas | 0.013674279 |
| OTU23 | Proteobacteria;Gammaproteobacteria;Xanthomonadales;Xanthomonadaceae;Thermomonas | 0.04956926 |
| OTU24 | Proteobacteria;Gammaproteobacteria;Xanthomonadales;Xanthomonadaceae;Stenotrophomonas | 0.451251196 |
| OTU25 | Proteobacteria;Gammaproteobacteria;Xanthomonadales;Xanthomonadaceae;Dokdonella | 0.006837139 |
| OTU26 | Proteobacteria;Gammaproteobacteria;Pseudomonadales;Pseudomonadaceae;Pseudomonas | 0.015383564 |
| OTU27 | Proteobacteria;Gammaproteobacteria;Pseudomonadales;Pseudomonadaceae;Pseudomonas | 0.005127855 |
| OTU28 | Proteobacteria;Gammaproteobacteria;Pseudomonadales;Pseudomonadaceae;Pseudomonas | 0.015383564 |
| OTU29 | Proteobacteria;Gammaproteobacteria;Pseudomonadales;Pseudomonadaceae;Pseudomonas | 0.008546424 |
| OTU30 | Proteobacteria;Alphaproteobacteria;Sphingomonadales;Sphingomonadaceae;Sphingomonas | 22.7574183 |
| OTU31 | Proteobacteria;Alphaproteobacteria;Sphingomonadales;Sphingomonadaceae;Sphingomonas | 0.032476412 |
| OTU32 | Proteobacteria;Alphaproteobacteria;Sphingomonadales;Sphingomonadaceae;Sphingomonas | 0.013674279 |
| OTU33 | Proteobacteria;Alphaproteobacteria;Sphingomonadales;Sphingomonadaceae | 0.006837139 |
| OTU34 | Proteobacteria;Alphaproteobacteria;Rhodospirillales;Acetobacteraceae;Acidiphilium | 0.059824969 |
| OTU35 | Proteobacteria;Alphaproteobacteria;Rhodospirillales;Acetobacteraceae | 0.480309039 |
| OTU36 | Proteobacteria;Alphaproteobacteria;Rhizobiales;Hyphomicrobiaceae;Hyphomicrobium | 0.011964994 |
| OTU37 | Proteobacteria;Alphaproteobacteria;Rhodobacterales;Rhodobacteraceae;Paracoccus | 0.006837139 |
| OTU38 | Proteobacteria;Alphaproteobacteria;Caulobacterales;Caulobacteraceae;Brevundimonas | 0.035894982 |
| OTU39 | Proteobacteria;Alphaproteobacteria;Caulobacterales;Caulobacteraceae;Brevundimonas | 0.230753453 |
| OTU40 | Proteobacteria;Alphaproteobacteria;Rhizobiales;Bradyrhizobiaceae; Bosea | 0.008546424 |
| OTU41 | Proteobacteria;Alphaproteobacteria;Rhizobiales;Methylobacteriaceae;Microvirga | 0.011964994 |
| OTU42 | Proteobacteria;Alphaproteobacteria;Rhizobiales;Methylobacteriaceae;Methylobacterium | 0.037604266 |
| OTU43 | Proteobacteria;Alphaproteobacteria;Rhizobiales;Methylobacteriaceae;Methylobacterium | 0.017092848 |
| OTU44 | Proteobacteria;Deltaproteobacteria;Bdellovibrionales;Bacteriovoracaceae;Peredibacter | 0.020511418 |
| OTU45 | Firmicutes;Clostridia;Clostridiales;Tissierellaceae;Anaerococcus | 0.068371393 |
| OTU46 | Firmicutes;Bacilli;Bacillales;Paenibacillaceae;Paenibacillus | 0.082045672 |
| OTU47 | Firmicutes;Bacilli;Bacillales;Paenibacillaceae;Paenibacillus | 0.047859975 |
| OTU48 | Firmicutes;Bacilli;Bacillales;Paenibacillaceae;Paenibacillus | 0.082045672 |
| OTU49 | Firmicutes;Bacilli;Bacillales;Paenibacillaceae;Paenibacillus | 0.107684945 |
| OTU50 | Firmicutes;Bacilli;Bacillales;Paenibacillaceae;Brevibacillus | 0.109394229 |
| OTU51 | Firmicutes;Bacilli;Bacillales;Paenibacillaceae;Brevibacillus | 0.497401887 |
| OTU52 | Firmicutes;Bacilli;Lactobacillales;Streptococcaceae;Streptococcus | 0.005127855 |
| OTU53 | Firmicutes;Bacilli;Lactobacillales;Streptococcaceae;Streptococcus | 0.10255709 |
| OTU54 | Firmicutes;Bacilli;Lactobacillales;Carnobacteriaceae;Granulicatella | 0.061534254 |
| OTU55 | Firmicutes;Bacilli;Bacillales;Paenibacillaceae;Aneurinibacillus | 0.082045672 |
| OTU56 | Firmicutes;Bacilli;Bacillales;Bacillaceae;Geobacillus | 0.015383564 |
| OTU57 | Firmicutes;Bacilli;Bacillales;Bacillaceae;Bacillus | 0.153835635 |
| OTU58 | Firmicutes;Bacilli;Bacillales;Planococcaceae;Planomicrobium | 0.037604266 |
| OTU59 | Firmicutes;Bacilli;Gemellales;Gemellaceae;Gemella | 0.030767127 |
| OTU60 | Firmicutes;Bacilli;Bacillales;Staphylococcaceae;Staphylococcus | 27.40496376 |
| OTU61 | Firmicutes;Bacilli;Bacillales;Staphylococcaceae;Staphylococcus | 0.035894982 |
| OTU62 | Firmicutes;Bacilli;Bacillales;Bacillaceae | 0.011964994 |
| OTU63 | Firmicutes;Bacilli;Bacillales;Alicyclobacillaceae;Alicyclobacillus | 0.129905647 |
| OTU64 | Firmicutes;Clostridia;Clostridiales;Veillonellaceae;Veillonella | 0.083754957 |
| OTU65 | Firmicutes;Clostridia;Clostridiales;Lachnospiraceae;Oribacterium | 0.018802133 |
| OTU66 | Firmicutes;Clostridia;Clostridiales;Ruminococcaceae;Faecalibacterium | 0.005127855 |
| OTU67 | Actinobacteria;Actinobacteria;Actinomycetales;Mycobacteriaceae;Mycobacterium | 0.011964994 |
| OTU68 | Actinobacteria;Actinobacteria;Actinomycetales;Nocardiaceae;Rhodococcus | 0.011964994 |
| OTU69 | Actinobacteria;Actinobacteria;Actinomycetales;Nocardiaceae;Rhodococcus | 0.00341857 |
| OTU70 | Actinobacteria;Actinobacteria;Actinomycetales;Corynebacteriaceae;Corynebacterium | 0.080336387 |
| OTU71 | Actinobacteria;Actinobacteria;Actinomycetales;Corynebacteriaceae;Corynebacterium | 0.10255709 |
| OTU72 | Actinobacteria;Actinobacteria;Actinomycetales;Intrasporangiaceae;Janibacter | 0.006837139 |
| OTU73 | Actinobacteria;Actinobacteria;Actinomycetales;Micrococcaceae | 0.015383564 |
| OTU74 | Actinobacteria;Actinobacteria;Actinomycetales;Streptomycetaceae;Streptomyces | 0.011964994 |
| OTU75 | Actinobacteria;Actinobacteria;Bifidobacteriales;Bifidobacteriaceae;Gardnerella | 0.008546424 |
| OTU76 | Actinobacteria;Actinobacteria;Actinomycetales;Thermomonosporaceae;Actinomadura | 0.04956926 |
| OTU77 | Actinobacteria;Actinobacteria;Actinomycetales;Propionibacteriaceae;Propionibacterium | 0.083754957 |
| OTU78 | Bacteroidetes;Bacteroidia;Bacteroidales;Porphyromonadaceae;Dysgonomonas | 0.001709285 |
| OTU79 | Bacteroidetes;Bacteroidia;Bacteroidales;Porphyromonadaceae;Dysgonomonas | 0.047859975 |
| OTU80 | Bacteroidetes;Bacteroidia;Bacteroidales;Porphyromonadaceae | 0.005127855 |
| OTU81 | Bacteroidetes;Bacteroidia;Bacteroidales;Prevotellaceae;Prevotella | 0.008546424 |
| OTU82 | Bacteroidetes;Bacteroidia;Bacteroidales;Prevotellaceae;Prevotella | 0.00341857 |
| OTU83 | Bacteroidetes;Bacteroidia;Bacteroidales;Paraprevotellaceae;Alloprevotella | 0.018802133 |
| OTU84 | Bacteroidetes;Bacteroidia;Bacteroidales;Paraprevotellaceae; | 0.013674279 |
| OTU85 | Bacteroidetes;Sphingobacteriia;Sphingobacteriales;Sphingobacteriaceae;Sphingobacterium | 0.04956926 |
| OTU86 | Bacteroidetes;Sphingobacteriia;Sphingobacteriales;Sphingobacteriaceae;Sphingobacterium | 0.011964994 |
| OTU87 | Bacteroidetes;Saprospirae;Saprospirales;Chitinophagaceae;Flavisolibacter | 0.04956926 |
| OTU88 | Bacteroidetes;Saprospirae;Saprospirales;Chitinophagaceae | 0.010255709 |
| OTU89 | Bacteroidetes;Flavobacteriia;Flavobacteriales;Flavobacteriaceae;Flavobacterium | 0.008546424 |
| OTU90 | Bacteroidetes;Flavobacteriia;Flavobacteriales;Flavobacteriaceae;Gillisia | 0.029057842 |
| OTU91 | Bacteroidetes;Flavobacteriia;Flavobacteriales;Weeksellaceae;Chryseobacterium | 0.059824969 |
| OTU92 | Fusobacteria;Fusobacteriia;Fusobacteriales;Fusobacteriaceae;Fusobacterium | 0.059824969 |
| OTU93 | Acidobacteria;Acidobacteria-Gp6 | 0.015383564 |
| OTU94 | Acidobacteria;Acidobacteria_Gp4/Chloracidobacteria;RB41 | 0.015383564 |
| OTU95 | Acidobacteria;Acidobacteria_Gp4/Chloracidobacteria;RB41 | 0.022220703 |
| OTU96 | Thermi;Deinococci;Deinococcales;Deinococcaceae;Deinococcus | 0.008546424 |
| OTU97 | Planctomycetes;Phycisphaerae;Tepidisphaerales;Tepidisphaeraceae;Tepidisphaera | 0.00341857 |
| OTU98 | Verrucomicrobia; | 0.042732121 |
| OTU99 | Nitrospirae;Nitrospira;Nitrospirales;Leptospirillaceae;Leptospirillum | 0.011964994 |

Table S3. Community profiling based on metagenomic sequencing of bacterial 16S rRNA genes. OTU abundances were given for original community (OMG)

| OTU | Taxon | Relative abundance |
| --- | --- | --- |
| Otu1 | Bacteria;ZB3;Rs-J96 | 0.010690995 |
| Otu2 | Bacteria;ZB3;Rs-J96 | 0.121164606 |
| Otu3 | Bacteria;Verrucomicrobia;Spartobacteria;Chthoniobacterales;Chthoniobacteraceae;heteroC45_4W | 0.010690995 |
| Otu4 | Bacteria;Verrucomicrobia;Opitutae;Opitutales;Opitutaceae;Opitutus | 0.042763978 |
| Otu5 | Bacteria;Verrucomicrobia;Opitutae;HA64 | 0.406257796 |
| Otu6 | Bacteria;Verrucomicrobia;Opitutae;HA64 | 0.042763978 |
| Otu7 | Bacteria;Verrucomicrobia;Opitutae | 0.064145968 |
| Otu8 | Bacteria;Verrucomicrobia;Methylacidiphilae;Methylacidiphilales;LD19 | 0.014254659 |
| Otu9 | Bacteria;Tenericutes;Mollicutes;RF39 | 0.00712733 |
| Otu10 | Bacteria;Tenericutes;Mollicutes;Mycoplasmatales;Mycoplasmataceae | 0.021381989 |
| Otu11 | Bacteria;Tenericutes;Mollicutes;Mycoplasmatales;Mycoplasmataceae | 0.808951926 |
| Otu12 | Bacteria;Tenericutes;Mollicutes;Mycoplasmatales;Mycoplasmataceae | 0.135419265 |
| Otu13 | Bacteria;Synergistetes;Synergistia;Synergistales;Synergistaceae;Candidatus_Tammella | 0.014254659 |
| Otu14 | Bacteria;Synergistetes;Synergistia;Synergistales;Synergistaceae;Candidatus_Tammella | 0.071273297 |
| Otu15 | Bacteria;Synergistetes;Synergistia;Synergistales;Dethiosulfovibrionaceae;TG5 | 0.253020206 |
| Otu16 | Bacteria;Synergistetes;Synergistia;Synergistales;Dethiosulfovibrionaceae;TG5 | 0.067709633 |
| Otu17 | Bacteria;Synergistetes;Synergistia;Synergistales;Dethiosulfovibrionaceae;TG5 | 0.060582303 |
| Otu18 | Bacteria;Synergistetes;Synergistia;Synergistales;Dethiosulfovibrionaceae;TG5 | 0.049891308 |
| Otu19 | Bacteria;Spirochaetes;Spirochaetes;Spirochaetales;Spirochaetaceae;za29 | 0.057018638 |
| Otu20 | Bacteria;Spirochaetes;Spirochaetes;Spirochaetales;Spirochaetaceae;za29 | 0.010690995 |
| Otu21 | Bacteria;Spirochaetes;Spirochaetes;Spirochaetales;Spirochaetaceae;Treponema | 0.97288051 |
| Otu22 | Bacteria;Spirochaetes;Spirochaetes;Spirochaetales;Spirochaetaceae;Treponema | 7.587042515 |
| Otu23 | Bacteria;Spirochaetes;Spirochaetes;Spirochaetales;Spirochaetaceae;Treponema | 0.438330779 |
| Otu24 | Bacteria;Spirochaetes;Spirochaetes;Spirochaetales;Spirochaetaceae;Treponema | 0.228074552 |
| Otu25 | Bacteria;Spirochaetes;Spirochaetes;Spirochaetales;Spirochaetaceae;Treponema | 2.159580913 |
| Otu26 | Bacteria;Spirochaetes;Spirochaetes;Spirochaetales;Spirochaetaceae;Treponema | 0.010690995 |
| Otu27 | Bacteria;Spirochaetes;Spirochaetes;Spirochaetales;Spirochaetaceae;Treponema | 0.306475179 |
| Otu28 | Bacteria;Spirochaetes;Spirochaetes;Spirochaetales;Spirochaetaceae;Treponema | 0.577313709 |
| Otu29 | Bacteria;Spirochaetes;Spirochaetes;Spirochaetales;Spirochaetaceae;Treponema | 0.384875806 |
| Otu30 | Bacteria;Spirochaetes;Spirochaetes;Spirochaetales;Spirochaetaceae;Treponema | 1.660667831 |
| Otu31 | Bacteria;Spirochaetes;Spirochaetes;Spirochaetales;Spirochaetaceae;Treponema | 0.377748477 |
| Otu32 | Bacteria;Spirochaetes;Spirochaetes;Spirochaetales;Spirochaetaceae;Treponema | 2.002779659 |
| Otu33 | Bacteria;Spirochaetes;Spirochaetes;Spirochaetales;Spirochaetaceae;Treponema | 0.028509319 |
| Otu34 | Bacteria;Spirochaetes;Spirochaetes;Spirochaetales;Spirochaetaceae;Treponema | 0.032072984 |
| Otu35 | Bacteria;Spirochaetes;Spirochaetes;Spirochaetales;Spirochaetaceae;Treponema | 0.064145968 |
| Otu36 | Bacteria;Spirochaetes;Spirochaetes;Spirochaetales;Spirochaetaceae;Treponema | 0.00712733 |
| Otu37 | Bacteria;Spirochaetes;Spirochaetes;Spirochaetales;Spirochaetaceae;Treponema | 1.190264068 |
| Otu38 | Bacteria;Spirochaetes;Spirochaetes;Spirochaetales;Spirochaetaceae;Treponema | 1.393392965 |
| Otu39 | Bacteria;Spirochaetes;Spirochaetes;Spirochaetales;Spirochaetaceae;Treponema | 0.324293503 |
| Otu40 | Bacteria;Spirochaetes;Spirochaetes;Spirochaetales;Spirochaetaceae;Treponema | 0.081964292 |
| Otu41 | Bacteria;Spirochaetes;Spirochaetes;Spirochaetales;Spirochaetaceae;Treponema | 0.096218952 |
| Otu42 | Bacteria;Spirochaetes;Spirochaetes;Spirochaetales;Spirochaetaceae;Treponema | 0.495349417 |
| Otu43 | Bacteria;Spirochaetes;Spirochaetes;Spirochaetales;Spirochaetaceae;Treponema | 0.655714337 |
| Otu44 | Bacteria;Spirochaetes;Spirochaetes;Spirochaetales;Spirochaetaceae;Treponema | 0.014254659 |
| Otu45 | Bacteria;Spirochaetes;Spirochaetes;Spirochaetales;Spirochaetaceae;Treponema | 0.114037276 |
| Otu46 | Bacteria;Spirochaetes;Spirochaetes;Spirochaetales;Spirochaetaceae;Treponema | 0.238765546 |
| Otu47 | Bacteria;Spirochaetes;Spirochaetes;Spirochaetales;Spirochaetaceae;Treponema | 0.128291935 |
| Otu48 | Bacteria;Spirochaetes;Spirochaetes;Spirochaetales;Spirochaetaceae;Treponema | 0.673532661 |
| Otu49 | Bacteria;Spirochaetes;Spirochaetes;Spirochaetales;Spirochaetaceae;Treponema | 1.083354121 |
| Otu50 | Bacteria;Spirochaetes;Spirochaetes;Spirochaetales;Spirochaetaceae;Treponema | 0.163928584 |
| Otu51 | Bacteria;Spirochaetes;Spirochaetes;Spirochaetales;Spirochaetaceae;Treponema | 0.167492249 |
| Otu52 | Bacteria;Spirochaetes;Spirochaetes;Spirochaetales;Spirochaetaceae;Treponema | 0.627205018 |
| Otu53 | Bacteria;Spirochaetes;Spirochaetes;Spirochaetales;Spirochaetaceae;Treponema | 0.491785752 |
| Otu54 | Bacteria;Spirochaetes;Spirochaetes;Spirochaetales;Spirochaetaceae;Treponema | 0.260147536 |
| Otu55 | Bacteria;Spirochaetes;Spirochaetes;Spirochaetales;Spirochaetaceae;Treponema | 0.395566801 |
| Otu56 | Bacteria;Spirochaetes;Spirochaetes;Spirochaetales;Spirochaetaceae;Treponema | 4.148105912 |
| Otu57 | Bacteria;Spirochaetes;Spirochaetes;Spirochaetales;Spirochaetaceae;Treponema | 0.039200314 |
| Otu58 | Bacteria;Spirochaetes;Spirochaetes;Spirochaetales;Spirochaetaceae;Treponema | 0.049891308 |
| Otu59 | Bacteria;Spirochaetes;Spirochaetes;Spirochaetales;Spirochaetaceae;Treponema | 0.196001568 |
| Otu60 | Bacteria;Spirochaetes;Spirochaetes;Spirochaetales;Spirochaetaceae;Treponema | 0.035636649 |
| Otu61 | Bacteria;Spirochaetes;Spirochaetes;Spirochaetales;Spirochaetaceae;Treponema | 0.121164606 |
| Otu62 | Bacteria;Spirochaetes;Spirochaetes;Spirochaetales;Spirochaetaceae;Treponema | 0.235201882 |
| Otu63 | Bacteria;Spirochaetes;Spirochaetes;Spirochaetales;Spirochaetaceae;Treponema | 0.213819892 |
| Otu64 | Bacteria;Spirochaetes;Spirochaetes;Spirochaetales;Spirochaetaceae;Treponema | 0.017818324 |
| Otu65 | Bacteria;Spirochaetes;Spirochaetes;Spirochaetales;Spirochaetaceae;Treponema | 0.192437903 |
| Otu66 | Bacteria;Spirochaetes;Spirochaetes;Spirochaetales;Spirochaetaceae;Treponema | 0.231638217 |
| Otu67 | Bacteria;Spirochaetes;Spirochaetes;Spirochaetales;Spirochaetaceae;Treponema | 0.413385125 |
| Otu68 | Bacteria;Spirochaetes;Spirochaetes;Spirochaetales;Spirochaetaceae;Treponema | 0.645023342 |
| Otu69 | Bacteria;Spirochaetes;Spirochaetes;Spirochaetales;Spirochaetaceae;Treponema | 0.680659991 |
| Otu70 | Bacteria;Spirochaetes;Spirochaetes;Spirochaetales;Spirochaetaceae;Treponema | 1.468229928 |
| Otu71 | Bacteria;Spirochaetes;Spirochaetes;Spirochaetales;Spirochaetaceae;Treponema | 0.185310573 |
| Otu72 | Bacteria;Spirochaetes;Spirochaetes;Spirochaetales;Spirochaetaceae;Treponema | 5.042585795 |
| Otu73 | Bacteria;Spirochaetes;Spirochaetes;Spirochaetales;Spirochaetaceae;Treponema | 2.783222266 |
| Otu74 | Bacteria;Spirochaetes;Spirochaetes;Spirochaetales;Spirochaetaceae;Treponema | 1.970706675 |
| Otu75 | Bacteria;Spirochaetes;Spirochaetes;Spirochaetales;Spirochaetaceae | 0.024945654 |
| Otu76 | Bacteria;Spirochaetes;Spirochaetes;Spirochaetales;Spirochaetaceae | 0.114037276 |
| Otu77 | Bacteria;Spirochaetes;Leptospirae;Leptospirales;Sediment-4;SJA-88 | 0.017818324 |
| Otu78 | Bacteria;Proteobacteria;Gammaproteobacteria;Pseudomonadales;Pseudomonadaceae;Pseudomonas | 0.039200314 |
| Otu79 | Bacteria;Proteobacteria;Gammaproteobacteria;Pseudomonadales;Pseudomonadaceae;Pseudomonas | 0.017818324 |
| Otu80 | Bacteria;Proteobacteria;Gammaproteobacteria;Pseudomonadales;Pseudomonadaceae;Pseudomonas | 0.024945654 |
| Otu81 | Bacteria;Proteobacteria;Gammaproteobacteria;Pseudomonadales;Moraxellaceae;Acinetobacter | 0.014254659 |
| Otu82 | Bacteria;Proteobacteria;Gammaproteobacteria;Enterobacteriales;Enterobacteriaceae;Escherichia | 0.010690995 |
| Otu83 | Bacteria;Proteobacteria;Gammaproteobacteria;Enterobacteriales;Enterobacteriaceae | 0.017818324 |
| Otu84 | Bacteria;Proteobacteria;Gammaproteobacteria;Enterobacteriales;Enterobacteriaceae | 0.734114964 |
| Otu85 | Bacteria;Proteobacteria;Epsilonproteobacteria;Campylobacterales;Helicobacteraceae;Helicobacter | 0.014254659 |
| Otu86 | Bacteria;Proteobacteria;Epsilonproteobacteria;Campylobacterales | 0.317166174 |
| Otu87 | Bacteria;Proteobacteria;Deltaproteobacteria;Spirobacillales | 0.035636649 |
| Otu88 | Bacteria;Proteobacteria;Deltaproteobacteria;Myxococcales | 0.032072984 |
| Otu89 | Bacteria;Proteobacteria;Deltaproteobacteria;Desulfovibrionales;Desulfovibrionaceae;Desulfovibrio | 1.318556003 |
| Otu90 | Bacteria;Proteobacteria;Deltaproteobacteria;Desulfovibrionales;Desulfovibrionaceae | 0.024945654 |
| Otu91 | Bacteria;Proteobacteria;Deltaproteobacteria;Desulfovibrionales;Desulfovibrionaceae | 0.00712733 |
| Otu92 | Bacteria;Proteobacteria;Deltaproteobacteria;Desulfovibrionales;Desulfovibrionaceae | 0.092655287 |
| Otu93 | Bacteria;Proteobacteria;Deltaproteobacteria;Desulfovibrionales;Desulfovibrionaceae | 0.021381989 |
| Otu94 | Bacteria;Proteobacteria;Deltaproteobacteria;Desulfovibrionales;Desulfovibrionaceae | 0.149673925 |
| Otu95 | Bacteria;Proteobacteria;Deltaproteobacteria;Desulfovibrionales;Desulfovibrionaceae | 0.085527957 |
| Otu96 | Bacteria;Proteobacteria;Deltaproteobacteria;Desulfovibrionales;Desulfovibrionaceae | 0.078400627 |
| Otu97 | Bacteria;Proteobacteria;Deltaproteobacteria;Desulfovibrionales;Desulfovibrionaceae | 0.00712733 |
| Otu98 | Bacteria;Proteobacteria;Deltaproteobacteria;Desulfovibrionales;Desulfovibrionaceae | 0.042763978 |
| Otu99 | Bacteria;Proteobacteria;Deltaproteobacteria;Desulfovibrionales;Desulfovibrionaceae | 0.064145968 |
| Otu100 | Bacteria;Proteobacteria;Deltaproteobacteria;Desulfovibrionales;Desulfovibrionaceae | 0.003563665 |
| Otu101 | Bacteria;Proteobacteria;Deltaproteobacteria;Desulfarculales;Desulfarculaceae | 0.106909946 |
| Otu102 | Bacteria;Proteobacteria;Betaproteobacteria;Rhodocyclales;Rhodocyclaceae;TS34 | 1.350628987 |
| Otu103 | Bacteria;Proteobacteria;Betaproteobacteria;Rhodocyclales;Rhodocyclaceae;TS34 | 2.644239336 |
| Otu104 | Bacteria;Proteobacteria;Betaproteobacteria;Rhodocyclales;Rhodocyclaceae;TS34 | 0.00712733 |
| Otu105 | Bacteria;Proteobacteria;Betaproteobacteria;Rhodocyclales;Rhodocyclaceae;Propionivibrio | 0.41694879 |
| Otu106 | Bacteria;Proteobacteria;Betaproteobacteria;Rhodocyclales;Rhodocyclaceae | 0.049891308 |
| Otu107 | Bacteria;Proteobacteria;Betaproteobacteria;Burkholderiales;Comamonadaceae;Delftia | 0.003563665 |
| Otu108 | Bacteria;Proteobacteria;Betaproteobacteria;Burkholderiales;Burkholderiaceae;Burkholderia | 0.017818324 |
| Otu109 | Bacteria;Proteobacteria;Betaproteobacteria;Burkholderiales;Burkholderiaceae;Burkholderia | 0.042763978 |
| Otu110 | Bacteria;Proteobacteria;Alphaproteobacteria;Rickettsiales | 0.053454973 |
| Otu111 | Bacteria;Proteobacteria;Alphaproteobacteria;Rickettsiales | 0.958625851 |
| Otu112 | Bacteria;Proteobacteria;Alphaproteobacteria;Rickettsiales | 1.019208154 |
| Otu113 | Bacteria;Proteobacteria;Alphaproteobacteria;Rickettsiales | 0.106909946 |
| Otu114 | Bacteria;Proteobacteria;Alphaproteobacteria;Rickettsiales | 0.024945654 |
| Otu115 | Bacteria;Proteobacteria;Alphaproteobacteria;Rickettsiales | 0.067709633 |
| Otu116 | Bacteria;Proteobacteria;Alphaproteobacteria;Rickettsiales | 0.014254659 |
| Otu117 | Bacteria;Proteobacteria;Alphaproteobacteria;Rickettsiales | 0.035636649 |
| Otu118 | Bacteria;Proteobacteria;Alphaproteobacteria;Rickettsiales | 0.042763978 |
| Otu119 | Bacteria;Proteobacteria;Alphaproteobacteria;Rickettsiales | 0.021381989 |
| Otu120 | Bacteria;Proteobacteria;Alphaproteobacteria;Rickettsiales | 0.256583871 |
| Otu121 | Bacteria;Proteobacteria;Alphaproteobacteria;Rickettsiales | 0.024945654 |
| Otu122 | Bacteria;Proteobacteria;Alphaproteobacteria;Rickettsiales | 0.028509319 |
| Otu123 | Bacteria;Proteobacteria;Alphaproteobacteria;Rickettsiales | 0.021381989 |
| Otu124 | Bacteria;Proteobacteria;Alphaproteobacteria;Rickettsiales | 0.00712733 |
| Otu125 | Bacteria;Proteobacteria;Alphaproteobacteria;Rickettsiales | 3.271444353 |
| Otu126 | Bacteria;Proteobacteria;Alphaproteobacteria;RF32 | 0.014254659 |
| Otu127 | Bacteria;Proteobacteria;Alphaproteobacteria;Caulobacterales;Caulobacteraceae | 0.00712733 |
| Otu128 | Bacteria;Proteobacteria;Alphaproteobacteria | 0.00712733 |
| Otu129 | Bacteria;Proteobacteria;Alphaproteobacteria | 0.024945654 |
| Otu130 | Bacteria;Proteobacteria | 0.021381989 |
| Otu131 | Bacteria;Proteobacteria | 0.021381989 |
| Otu132 | Bacteria;Proteobacteria | 0.392003136 |
| Otu133 | Bacteria;Proteobacteria | 0.021381989 |
| Otu134 | Bacteria;Proteobacteria | 0.213819892 |
| Otu135 | Bacteria;Proteobacteria | 0.042763978 |
| Otu136 | Bacteria;Proteobacteria | 0.224510887 |
| Otu137 | Bacteria;Planctomycetes;vadinHA49;PeHg47 | 0.014254659 |
| Otu138 | Bacteria;Planctomycetes;Planctomycetia;Pirellulales;Pirellulaceae | 0.039200314 |
| Otu139 | Bacteria;Planctomycetes;Planctomycetia;Pirellulales;Pirellulaceae | 0.078400627 |
| Otu140 | Bacteria;Planctomycetes;Planctomycetia;Pirellulales;Pirellulaceae | 0.035636649 |
| Otu141 | Bacteria;Planctomycetes;Planctomycetia;Pirellulales;Pirellulaceae | 0.014254659 |
| Otu142 | Bacteria;Planctomycetes;Planctomycetia;Pirellulales;Pirellulaceae | 0.089091622 |
| Otu143 | Bacteria;Firmicutes;Clostridia;Clostridiales;Ruminococcaceae;Sporobacter | 0.024945654 |
| Otu144 | Bacteria;Firmicutes;Clostridia;Clostridiales;Ruminococcaceae;Ruminococcus | 0.00712733 |
| Otu145 | Bacteria;Firmicutes;Clostridia;Clostridiales;Ruminococcaceae;Ruminococcus | 0.042763978 |
| Otu146 | Bacteria;Firmicutes;Clostridia;Clostridiales;Ruminococcaceae;Ruminococcus | 0.010690995 |
| Otu147 | Bacteria;Firmicutes;Clostridia;Clostridiales;Ruminococcaceae;Ruminococcus | 0.017818324 |
| Otu148 | Bacteria;Firmicutes;Clostridia;Clostridiales;Ruminococcaceae;Ruminococcus | 0.042763978 |
| Otu149 | Bacteria;Firmicutes;Clostridia;Clostridiales;Ruminococcaceae;Ruminococcus | 0.210256228 |
| Otu150 | Bacteria;Firmicutes;Clostridia;Clostridiales;Ruminococcaceae;Ruminococcus | 0.039200314 |
| Otu151 | Bacteria;Firmicutes;Clostridia;Clostridiales;Ruminococcaceae;Ruminococcus | 0.024945654 |
| Otu152 | Bacteria;Firmicutes;Clostridia;Clostridiales;Ruminococcaceae;Papillibacter | 0.010690995 |
| Otu153 | Bacteria;Firmicutes;Clostridia;Clostridiales;Ruminococcaceae;Papillibacter | 0.035636649 |
| Otu154 | Bacteria;Firmicutes;Clostridia;Clostridiales;Ruminococcaceae;Papillibacter | 0.032072984 |
| Otu155 | Bacteria;Firmicutes;Clostridia;Clostridiales;Ruminococcaceae;Ethanoligenens | 0.00712733 |
| Otu156 | Bacteria;Firmicutes;Clostridia;Clostridiales;Ruminococcaceae;Clostridium | 0.028509319 |
| Otu157 | Bacteria;Firmicutes;Clostridia;Clostridiales;Ruminococcaceae | 0.035636649 |
| Otu158 | Bacteria;Firmicutes;Clostridia;Clostridiales;Ruminococcaceae | 0.00712733 |
| Otu159 | Bacteria;Firmicutes;Clostridia;Clostridiales;Ruminococcaceae | 0.024945654 |
| Otu160 | Bacteria;Firmicutes;Clostridia;Clostridiales;Ruminococcaceae | 0.081964292 |
| Otu161 | Bacteria;Firmicutes;Clostridia;Clostridiales;Ruminococcaceae | 0.00712733 |
| Otu162 | Bacteria;Firmicutes;Clostridia;Clostridiales;Ruminococcaceae | 0.010690995 |
| Otu163 | Bacteria;Firmicutes;Clostridia;Clostridiales;Ruminococcaceae | 0.00712733 |
| Otu164 | Bacteria;Firmicutes;Clostridia;Clostridiales;Ruminococcaceae | 0.017818324 |
| Otu165 | Bacteria;Firmicutes;Clostridia;Clostridiales;Ruminococcaceae | 0.017818324 |
| Otu166 | Bacteria;Firmicutes;Clostridia;Clostridiales;Ruminococcaceae | 0.014254659 |
| Otu167 | Bacteria;Firmicutes;Clostridia;Clostridiales;Ruminococcaceae | 0.024945654 |
| Otu168 | Bacteria;Firmicutes;Clostridia;Clostridiales;Ruminococcaceae | 0.021381989 |
| Otu169 | Bacteria;Firmicutes;Clostridia;Clostridiales;Ruminococcaceae | 0.067709633 |
| Otu170 | Bacteria;Firmicutes;Clostridia;Clostridiales;Ruminococcaceae | 0.021381989 |
| Otu171 | Bacteria;Firmicutes;Clostridia;Clostridiales;Ruminococcaceae | 0.00712733 |
| Otu172 | Bacteria;Firmicutes;Clostridia;Clostridiales;Ruminococcaceae | 0.017818324 |
| Otu173 | Bacteria;Firmicutes;Clostridia;Clostridiales;Ruminococcaceae | 0.017818324 |
| Otu174 | Bacteria;Firmicutes;Clostridia;Clostridiales;Ruminococcaceae | 0.053454973 |
| Otu175 | Bacteria;Firmicutes;Clostridia;Clostridiales;Ruminococcaceae | 0.053454973 |
| Otu176 | Bacteria;Firmicutes;Clostridia;Clostridiales;Ruminococcaceae | 0.192437903 |
| Otu177 | Bacteria;Firmicutes;Clostridia;Clostridiales;Ruminococcaceae | 0.010690995 |
| Otu178 | Bacteria;Firmicutes;Clostridia;Clostridiales;Ruminococcaceae | 0.00712733 |
| Otu179 | Bacteria;Firmicutes;Clostridia;Clostridiales;Ruminococcaceae | 0.032072984 |
| Otu180 | Bacteria;Firmicutes;Clostridia;Clostridiales;Ruminococcaceae | 0.046327643 |
| Otu181 | Bacteria;Firmicutes;Clostridia;Clostridiales;Ruminococcaceae | 0.035636649 |
| Otu182 | Bacteria;Firmicutes;Clostridia;Clostridiales;Ruminococcaceae | 0.00712733 |
| Otu183 | Bacteria;Firmicutes;Clostridia;Clostridiales;Ruminococcaceae | 0.00712733 |
| Otu184 | Bacteria;Firmicutes;Clostridia;Clostridiales;Ruminococcaceae | 0.1318556 |
| Otu185 | Bacteria;Firmicutes;Clostridia;Clostridiales;Ruminococcaceae | 0.014254659 |
| Otu186 | Bacteria;Firmicutes;Clostridia;Clostridiales;Ruminococcaceae | 0.021381989 |
| Otu187 | Bacteria;Firmicutes;Clostridia;Clostridiales;Ruminococcaceae | 0.010690995 |
| Otu188 | Bacteria;Firmicutes;Clostridia;Clostridiales;Ruminococcaceae | 0.00712733 |
| Otu189 | Bacteria;Firmicutes;Clostridia;Clostridiales;Ruminococcaceae | 0.060582303 |
| Otu190 | Bacteria;Firmicutes;Clostridia;Clostridiales;Ruminococcaceae | 0.014254659 |
| Otu191 | Bacteria;Firmicutes;Clostridia;Clostridiales;Ruminococcaceae | 0.00712733 |
| Otu192 | Bacteria;Firmicutes;Clostridia;Clostridiales;Ruminococcaceae | 0.032072984 |
| Otu193 | Bacteria;Firmicutes;Clostridia;Clostridiales;Ruminococcaceae | 0.032072984 |
| Otu194 | Bacteria;Firmicutes;Clostridia;Clostridiales;Ruminococcaceae | 0.103346281 |
| Otu195 | Bacteria;Firmicutes;Clostridia;Clostridiales;Peptococcaceae;Dehalobacter | 0.010690995 |
| Otu196 | Bacteria;Firmicutes;Clostridia;Clostridiales;Mogibacteriaceae;Anaerovorax | 0.021381989 |
| Otu197 | Bacteria;Firmicutes;Clostridia;Clostridiales;Mogibacteriaceae | 0.039200314 |
| Otu198 | Bacteria;Firmicutes;Clostridia;Clostridiales;Mogibacteriaceae | 0.024945654 |
| Otu199 | Bacteria;Firmicutes;Clostridia;Clostridiales;Mogibacteriaceae | 0.388439471 |
| Otu200 | Bacteria;Firmicutes;Clostridia;Clostridiales;Mogibacteriaceae | 0.067709633 |
| Otu201 | Bacteria;Firmicutes;Clostridia;Clostridiales;Mogibacteriaceae | 0.035636649 |
| Otu202 | Bacteria;Firmicutes;Clostridia;Clostridiales;Mogibacteriaceae | 0.014254659 |
| Otu203 | Bacteria;Firmicutes;Clostridia;Clostridiales;Mogibacteriaceae | 0.074836962 |
| Otu204 | Bacteria;Firmicutes;Clostridia;Clostridiales;Mogibacteriaceae | 0.459712769 |
| Otu205 | Bacteria;Firmicutes;Clostridia;Clostridiales;Mogibacteriaceae | 0.017818324 |
| Otu206 | Bacteria;Firmicutes;Clostridia;Clostridiales;Lachnospiraceae;Clostridium; | 0.049891308 |
| Otu207 | Bacteria;Firmicutes;Clostridia;Clostridiales;Lachnospiraceae;Clostridium; | 0.021381989 |
| Otu208 | Bacteria;Firmicutes;Clostridia;Clostridiales;Lachnospiraceae;Clostridium; | 0.00712733 |
| Otu209 | Bacteria;Firmicutes;Clostridia;Clostridiales;Lachnospiraceae;Clostridium; | 0.010690995 |
| Otu210 | Bacteria;Firmicutes;Clostridia;Clostridiales;Lachnospiraceae;Clostridium; | 0.049891308 |
| Otu211 | Bacteria;Firmicutes;Clostridia;Clostridiales;Lachnospiraceae;Clostridium; | 1.635722177 |
| Otu212 | Bacteria;Firmicutes;Clostridia;Clostridiales;Lachnospiraceae;Clostridium | 0.024945654 |
| Otu213 | Bacteria;Firmicutes;Clostridia;Clostridiales;Lachnospiraceae;Clostridium | 0.010690995 |
| Otu214 | Bacteria;Firmicutes;Clostridia;Clostridiales;Lachnospiraceae;Clostridium | 0.017818324 |
| Otu215 | Bacteria;Firmicutes;Clostridia;Clostridiales;Lachnospiraceae | 0.057018638 |
| Otu216 | Bacteria;Firmicutes;Clostridia;Clostridiales;Lachnospiraceae | 0.00712733 |
| Otu217 | Bacteria;Firmicutes;Clostridia;Clostridiales;Lachnospiraceae | 1.935070026 |
| Otu218 | Bacteria;Firmicutes;Clostridia;Clostridiales;Clostridiaceae;Clostridium | 0.014254659 |
| Otu219 | Bacteria;Firmicutes;Clostridia;Clostridiales;Clostridiaceae;Clostridium | 0.028509319 |
| Otu220 | Bacteria;Firmicutes;Clostridia;Clostridiales;Clostridiaceae | 0.024945654 |
| Otu221 | Bacteria;Firmicutes;Clostridia;Clostridiales;Christensenellaceae | 0.017818324 |
| Otu222 | Bacteria;Firmicutes;Clostridia;Clostridiales;Christensenellaceae | 0.017818324 |
| Otu223 | Bacteria;Firmicutes;Clostridia;Clostridiales | 0.028509319 |
| Otu224 | Bacteria;Firmicutes;Clostridia;Clostridiales | 0.017818324 |
| Otu225 | Bacteria;Firmicutes;Clostridia;Clostridiales | 0.00712733 |
| Otu226 | Bacteria;Firmicutes;Clostridia;Clostridiales | 0.00712733 |
| Otu227 | Bacteria;Firmicutes;Clostridia;Clostridiales | 0.028509319 |
| Otu228 | Bacteria;Firmicutes;Clostridia;Clostridiales | 0.00712733 |
| Otu229 | Bacteria;Firmicutes;Clostridia;Clostridiales | 0.010690995 |
| Otu230 | Bacteria;Firmicutes;Clostridia;Clostridiales | 0.00712733 |
| Otu231 | Bacteria;Firmicutes;Clostridia;Clostridiales | 0.306475179 |
| Otu232 | Bacteria;Firmicutes;Clostridia;Clostridiales | 0.017818324 |
| Otu233 | Bacteria;Firmicutes;Clostridia;Clostridiales | 0.017818324 |
| Otu234 | Bacteria;Firmicutes;Clostridia;Clostridiales | 0.021381989 |
| Otu235 | Bacteria;Firmicutes;Clostridia;Clostridiales | 0.744805958 |
| Otu236 | Bacteria;Firmicutes;Clostridia;Clostridiales | 0.00712733 |
| Otu237 | Bacteria;Firmicutes;Clostridia;Clostridiales | 0.00712733 |
| Otu238 | Bacteria;Firmicutes;Clostridia;Clostridiales | 0.010690995 |
| Otu239 | Bacteria;Firmicutes;Clostridia;Clostridiales | 0.017818324 |
| Otu240 | Bacteria;Firmicutes;Clostridia;Clostridiales | 0.014254659 |
| Otu241 | Bacteria;Firmicutes;Clostridia;Clostridiales | 0.010690995 |
| Otu242 | Bacteria;Firmicutes;Clostridia;Clostridiales | 0.064145968 |
| Otu243 | Bacteria;Firmicutes;Clostridia;Clostridiales | 0.042763978 |
| Otu244 | Bacteria;Firmicutes;Clostridia;Clostridiales | 0.057018638 |
| Otu245 | Bacteria;Firmicutes;Clostridia;Clostridiales | 0.014254659 |
| Otu246 | Bacteria;Firmicutes;Clostridia;Clostridiales | 0.017818324 |
| Otu247 | Bacteria;Firmicutes;Clostridia;Clostridiales | 0.00712733 |
| Otu248 | Bacteria;Firmicutes;Clostridia;Clostridiales | 0.021381989 |
| Otu249 | Bacteria;Firmicutes;Clostridia;Clostridiales | 0.010690995 |
| Otu250 | Bacteria;Firmicutes;Clostridia;Clostridiales | 0.024945654 |
| Otu251 | Bacteria;Firmicutes;Clostridia;Clostridiales | 0.00712733 |
| Otu252 | Bacteria;Firmicutes;Clostridia;Clostridiales | 0.021381989 |
| Otu253 | Bacteria;Firmicutes;Clostridia;Clostridiales | 0.024945654 |
| Otu254 | Bacteria;Firmicutes;Clostridia;Clostridiales | 0.017818324 |
| Otu255 | Bacteria;Firmicutes;Clostridia;Clostridiales | 0.078400627 |
| Otu256 | Bacteria;Firmicutes;Clostridia | 0.010690995 |
| Otu257 | Bacteria;Firmicutes;Bacilli;Lactobacillales;Streptococcaceae;Streptococcus | 0.003563665 |
| Otu258 | Bacteria;Firmicutes;Bacilli;Lactobacillales;Streptococcaceae;Lactococcus | 0.032072984 |
| Otu259 | Bacteria;Firmicutes;Bacilli;Lactobacillales;Streptococcaceae;Lactococcus | 0.021381989 |
| Otu260 | Bacteria;Firmicutes;Bacilli;Lactobacillales;Streptococcaceae;Lactococcus | 1.122554435 |
| Otu261 | Bacteria;Firmicutes;Bacilli;Lactobacillales;Leuconostocaceae;Leuconostoc | 0.014254659 |
| Otu262 | Bacteria;Firmicutes;Bacilli;Lactobacillales;Enterococcaceae;Tetragenococcus | 0.032072984 |
| Otu263 | Bacteria;Firmicutes;Bacilli;Lactobacillales;Carnobacteriaceae;Granulicatella | 0.021381989 |
| Otu264 | Bacteria;Firmicutes;Bacilli;Bacillales;Planococcaceae;Planomicrobium | 0.003563665 |
| Otu265 | Bacteria;Firmicutes;Bacilli;Bacillales;Paenibacillaceae;Paenibacillus | 0.00712733 |
| Otu266 | Bacteria;Firmicutes;Bacilli;Bacillales;Paenibacillaceae;Paenibacillus | 0.046327643 |
| Otu267 | Bacteria;Firmicutes;Bacilli;Bacillales;Listeriaceae;Brochothrix | 0.042763978 |
| Otu268 | Bacteria;Firmicutes;Bacilli;Bacillales;Bacillaceae;Bacillus;Bacillus_cereus | 3.417554613 |
| Otu269 | Bacteria;Firmicutes;Bacilli;Bacillales;Bacillaceae;Bacillus | 0.092655287 |
| Otu270 | Bacteria;Firmicutes;Bacilli;Bacillales;Bacillaceae | 0.13898293 |
| Otu271 | Bacteria;Firmicutes | 0.010690995 |
| Otu272 | Bacteria;Firmicutes | 0.028509319 |
| Otu273 | Bacteria;Firmicutes | 0.010690995 |
| Otu274 | Bacteria;Firmicutes | 0.060582303 |
| Otu275 | Bacteria;Firmicutes | 0.024945654 |
| Otu276 | Bacteria;Firmicutes | 0.117600941 |
| Otu277 | Bacteria;Elusimicrobia;Endomicrobia | 0.017818324 |
| Otu278 | Bacteria;Elusimicrobia;Endomicrobia | 0.744805958 |
| Otu279 | Bacteria;Elusimicrobia;Endomicrobia | 0.509604077 |
| Otu280 | Bacteria;Elusimicrobia;Endomicrobia | 0.053454973 |
| Otu281 | Bacteria;Elusimicrobia;Endomicrobia | 1.931506361 |
| Otu282 | Bacteria;Elusimicrobia;Endomicrobia | 0.306475179 |
| Otu283 | Bacteria;Elusimicrobia;Endomicrobia | 0.947934856 |
| Otu284 | Bacteria;Elusimicrobia;Endomicrobia | 0.167492249 |
| Otu285 | Bacteria;Elusimicrobia;Endomicrobia | 0.00712733 |
| Otu286 | Bacteria;Elusimicrobia;Endomicrobia | 0.374184812 |
| Otu287 | Bacteria;Elusimicrobia;Endomicrobia | 2.487438081 |
| Otu288 | Bacteria;Elusimicrobia;Endomicrobia | 6.1580129 |
| Otu289 | Bacteria;Elusimicrobia;Endomicrobia | 0.081964292 |
| Otu290 | Bacteria;Bacteroidetes;Sphingobacteriia;Sphingobacteriales;Sphingobacteriaceae;Sphingobacterium | 0.003563665 |
| Otu291 | Bacteria;Bacteroidetes;Flavobacteriia;Flavobacteriales;Weeksellaceae;Chryseobacterium | 0.00712733 |
| Otu292 | Bacteria;Bacteroidetes;Flavobacteriia;Flavobacteriales;Weeksellaceae;Chryseobacterium | 0.003563665 |
| Otu293 | Bacteria;Bacteroidetes;Flavobacteriia;Flavobacteriales | 0.035636649 |
| Otu294 | Bacteria;Bacteroidetes;Bacteroidia;Bacteroidales;Rikenellaceae | 0.049891308 |
| Otu295 | Bacteria;Bacteroidetes;Bacteroidia;Bacteroidales;Rikenellaceae | 0.010690995 |
| Otu296 | Bacteria;Bacteroidetes;Bacteroidia;Bacteroidales;Rikenellaceae | 0.021381989 |
| Otu297 | Bacteria;Bacteroidetes;Bacteroidia;Bacteroidales;Porphyromonadaceae;Tannerella | 0.067709633 |
| Otu298 | Bacteria;Bacteroidetes;Bacteroidia;Bacteroidales;Porphyromonadaceae;Paludibacter | 0.049891308 |
| Otu299 | Bacteria;Bacteroidetes;Bacteroidia;Bacteroidales;Porphyromonadaceae;Paludibacter | 0.039200314 |
| Otu300 | Bacteria;Bacteroidetes;Bacteroidia;Bacteroidales;Porphyromonadaceae;Paludibacter | 0.032072984 |
| Otu301 | Bacteria;Bacteroidetes;Bacteroidia;Bacteroidales;Porphyromonadaceae;Dysgonomonas | 0.235201882 |
| Otu302 | Bacteria;Bacteroidetes;Bacteroidia;Bacteroidales;Porphyromonadaceae;Dysgonomonas | 0.00712733 |
| Otu303 | Bacteria;Bacteroidetes;Bacteroidia;Bacteroidales;Porphyromonadaceae;Dysgonomonas | 0.27083853 |
| Otu304 | Bacteria;Bacteroidetes;Bacteroidia;Bacteroidales;Porphyromonadaceae;Dysgonomonas | 0.021381989 |
| Otu305 | Bacteria;Bacteroidetes;Bacteroidia;Bacteroidales;Porphyromonadaceae;Dysgonomonas | 0.124728271 |
| Otu306 | Bacteria;Bacteroidetes;Bacteroidia;Bacteroidales;Porphyromonadaceae;Dysgonomonas | 0.010690995 |
| Otu307 | Bacteria;Bacteroidetes;Bacteroidia;Bacteroidales;Porphyromonadaceae;Candidatus_Azobacteroides | 1.525248566 |
| Otu308 | Bacteria;Bacteroidetes;Bacteroidia;Bacteroidales;Porphyromonadaceae;Candidatus_Azobacteroides | 0.135419265 |
| Otu309 | Bacteria;Bacteroidetes;Bacteroidia;Bacteroidales;Porphyromonadaceae;Candidatus_Azobacteroides | 0.210256228 |
| Otu310 | Bacteria;Bacteroidetes;Bacteroidia;Bacteroidales;Porphyromonadaceae;Candidatus_Azobacteroides | 0.121164606 |
| Otu311 | Bacteria;Bacteroidetes;Bacteroidia;Bacteroidales;Porphyromonadaceae;Candidatus_Azobacteroides | 0.231638217 |
| Otu312 | Bacteria;Bacteroidetes;Bacteroidia;Bacteroidales;Porphyromonadaceae;Candidatus_Azobacteroides | 0.028509319 |
| Otu313 | Bacteria;Bacteroidetes;Bacteroidia;Bacteroidales;Porphyromonadaceae;Candidatus_Azobacteroides | 0.010690995 |
| Otu314 | Bacteria;Bacteroidetes;Bacteroidia;Bacteroidales;Porphyromonadaceae;Candidatus_Azobacteroides | 0.021381989 |
| Otu315 | Bacteria;Bacteroidetes;Bacteroidia;Bacteroidales;Porphyromonadaceae;Candidatus_Azobacteroides | 0.064145968 |
| Otu316 | Bacteria;Bacteroidetes;Bacteroidia;Bacteroidales;Porphyromonadaceae;Candidatus_Azobacteroides | 0.046327643 |
| Otu317 | Bacteria;Bacteroidetes;Bacteroidia;Bacteroidales;Porphyromonadaceae;Candidatus_Azobacteroides | 0.049891308 |
| Otu318 | Bacteria;Bacteroidetes;Bacteroidia;Bacteroidales;Porphyromonadaceae;Candidatus_Azobacteroides | 0.00712733 |
| Otu319 | Bacteria;Bacteroidetes;Bacteroidia;Bacteroidales;Porphyromonadaceae;Candidatus_Azobacteroides | 1.984961334 |
| Otu320 | Bacteria;Bacteroidetes;Bacteroidia;Bacteroidales;Porphyromonadaceae;Candidatus_Azobacteroides | 1.796087096 |
| Otu321 | Bacteria;Bacteroidetes;Bacteroidia;Bacteroidales;Porphyromonadaceae | 0.267274865 |
| Otu322 | Bacteria;Bacteroidetes;Bacteroidia;Bacteroidales;Porphyromonadaceae | 0.15323759 |
| Otu323 | Bacteria;Bacteroidetes;Bacteroidia;Bacteroidales;Paraprevotellaceae;Prevotella | 0.021381989 |
| Otu324 | Bacteria;Bacteroidetes;Bacteroidia;Bacteroidales;p-2534-18B5 | 0.56305905 |
| Otu325 | Bacteria;Bacteroidetes;Bacteroidia;Bacteroidales;Odoribacteraceae;Odoribacter | 0.466840098 |
| Otu326 | Bacteria;Bacteroidetes;Bacteroidia;Bacteroidales | 0.021381989 |
| Otu327 | Bacteria;Bacteroidetes;Bacteroidia;Bacteroidales | 0.349239158 |
| Otu328 | Bacteria;Bacteroidetes;Bacteroidia;Bacteroidales | 0.057018638 |
| Otu329 | Bacteria;Bacteroidetes;Bacteroidia;Bacteroidales | 0.096218952 |
| Otu330 | Bacteria;Bacteroidetes;Bacteroidia;Bacteroidales | 0.014254659 |
| Otu331 | Bacteria;Bacteroidetes;Bacteroidia;Bacteroidales | 0.203128898 |
| Otu332 | Bacteria;Bacteroidetes;Bacteroidia;Bacteroidales | 0.174619579 |
| Otu333 | Bacteria;Bacteroidetes;Bacteroidia;Bacteroidales | 0.114037276 |
| Otu334 | Bacteria;Bacteroidetes;Bacteroidia;Bacteroidales | 0.010690995 |
| Otu335 | Bacteria;Bacteroidetes | 0.174619579 |
| Otu336 | Bacteria;Bacteroidetes | 0.021381989 |
| Otu337 | Bacteria;Bacteroidetes | 0.021381989 |
| Otu338 | Bacteria;Bacteroidetes | 0.00712733 |
| Otu339 | Bacteria;Actinobacteria;Coriobacteriia;Coriobacteriales;Coriobacteriaceae | 0.024945654 |
| Otu340 | Bacteria;Actinobacteria;Coriobacteriia;Coriobacteriales;Coriobacteriaceae | 0.188874238 |
| Otu341 | Bacteria;Actinobacteria;Coriobacteriia;Coriobacteriales;Coriobacteriaceae | 0.046327643 |
| Otu342 | Bacteria;Actinobacteria;Coriobacteriia;Coriobacteriales;Coriobacteriaceae | 0.15323759 |
| Otu343 | Bacteria;Actinobacteria;Coriobacteriia;Coriobacteriales;Coriobacteriaceae | 0.099782616 |
| Otu344 | Bacteria;Actinobacteria;Actinobacteria;Actinomycetales;Actinomycetaceae;Candidatus_Ancillula | 0.024945654 |
| Otu345 | Bacteria;Actinobacteria;Actinobacteria;Actinomycetales;Actinomycetaceae;Candidatus_Ancillula | 0.053454973 |
| Otu346 | Bacteria;Actinobacteria;Actinobacteria;Actinomycetales;Actinomycetaceae;Candidatus_Ancillula | 0.010690995 |
| Otu347 | Bacteria;Actinobacteria;Actinobacteria;Actinomycetales;Actinomycetaceae;Candidatus_Ancillula | 0.010690995 |
| Otu348 | Bacteria;Actinobacteria;Actinobacteria;Actinomycetales;Actinomycetaceae;Candidatus_Ancillula | 0.010690995 |
| Otu349 | Bacteria;Actinobacteria;Actinobacteria;Actinomycetales | 0.010690995 |
| Otu350 | Bacteria;Actinobacteria;Actinobacteria;Actinomycetales | 0.135419265 |
| Otu351 | Bacteria;Actinobacteria;Actinobacteria;Actinomycetales | 0.060582303 |
| Otu352 | Bacteria;Actinobacteria;Actinobacteria;Actinomycetales | 0.00712733 |
| Otu353 | Bacteria;Actinobacteria;Actinobacteria | 0.010690995 |
